# Supplementary material for: Protection of nascent DNA at stalled replication forks is mediated by phosphorylation of RIF1 intrinsically disordered region
Source: eLife. 2022 Apr 13;11:e75047. doi: 10.7554/eLife.75047 (PMC9007588; doi:10.7554/eLife.75047)
Supplement: Figure 1—source data 1. [file elife-75047-fig1-data1.docx]

**Figure 1 - source data 1. List of RIF1 protein homologs across representative species from the Animalia and Fungi kingdoms.**

| **Kingdom** | **Phylum (Animalia) /**  **Division (Fungi)** | **Class** | **Infraclass** | **Clade/**  **Superorder** | **Order** | **Genus & Species** | **Common name** | **RIF1**  **UniProt ID** | **N°**  **AA*** |
| --- | --- | --- | --- | --- | --- | --- | --- | --- | --- |
| Animalia | Chordata  (Vertebrates) | Mammalia | Placentalia | Euarchontoglires | Primates | *Homo sapiens* | Human | Q5UIP0-1 | 2472 |
|  |  |  |  |  |  | *Pan troglodytes* | Chimpanzee | A0A6D2Y1B4 | 2473 |
|  |  |  |  |  |  | Callithrix jacchus | Marmoset | F7I9U7 | 2463 |
|  |  |  |  |  | Rodentia | *Mus musculus* | Mouse | Q6PR54-1 | 2419 |
|  |  |  |  |  | Lagomorpha | *Oryctolagus cuniculus* | Rabbit | G1TYW6-1 | 2455 |
|  |  |  |  | Laurasiatheria | Artiodactyla | *Bos taurus* | Cow | E1BCK1 | 2313 |
|  |  |  |  |  | Perissodactyla | *Equus caballus* | Horse | A0A3Q2H5U1 | 2476 |
|  |  |  |  | Afrotheria | Proboscidea | *Loxodonta africana* | Elephant | G3T4D9 | 2441 |
|  |  |  |  | Xenarthra | Not included in the analysis. | | | |  |
|  |  |  | Marsupialia | / | Didelphimorphia | *Monodelphis domestica* | Opossum | K7E160 | 2408 |
|  |  |  | Monotremata | / | Monotremata | *Ornithorhynchus anatinus* | Platypus | F7BUB6 | 2455 |
|  |  | Aves (Birds) | / | / | Galliformes | *Gallus gallus* | Chicken | E1C2U2-1 | 2326 |
|  |  | Reptilia | / | / | Squamata | *Podarcis muralis* | Lizard | A0A670HUX7 | 1961 |
|  |  | Amphibia | / | / | Anura | *Xenopus laevis* | Xenopus or  African clawed frog | I6N3H5 | 2327 |
|  |  | Actinopterygii  (bony fishes) | / | / | Cypriniformes | *Danio rerio* | Zebrafish | B0UY57 | 2392 |
|  |  | Chondrichthyes (cartilaginous fishes) | / | / | Chimaeriformes | *Callorhinchus milii* | Shark | A0A4W3IQ02 | 2254 |
|  | Arthropoda (Invertebrates) | Insecta | / | / | Diptera | *Drosophila melanogaster* | Drosophila or  Fruit fly | Q9XZ34 | 1416 |
| Fungi | Ascomycota (Lower eukaryotes) | Saccharomycetes | / | / | Saccharomycetales | *Saccharomyces cerevisiae* | (Baker's) yeast | P29539 | 1916 |
|  |  | Schizosaccharomycetes | / | / | Schizosaccharomycetales | Schizosaccharomyces pombe | Fission yeast | Q96UP3 | 1400 |

*AA = amino acids
