# Supplementary material for: Protection of nascent DNA at stalled replication forks is mediated by phosphorylation of RIF1 intrinsically disordered region
Source: eLife. 2022 Apr 13;11:e75047. doi: 10.7554/eLife.75047 (PMC9007588; doi:10.7554/eLife.75047)
Supplement: Figure 1—source data 2. [file elife-75047-fig1-data2.docx]

**Figure 1 – source data 2. Alignment of peptides containing SQ/TQ motifs conserved between mouse and human RIF1 proteins across representative species from the Animalia and Fungi kingdoms.**

| **mRIF1 SQ** | **S66Q** | **S986Q** | **S1086Q-S1096Q** | **S1387Q** | **S1416Q** | **S1528Q** | **S1713Q** | **S1842Q** | **S2138Q** | **S2387Q** |
| --- | --- | --- | --- | --- | --- | --- | --- | --- | --- | --- |
| **hRIF1 SQ** | **S66Q** | **S989Q** | **S1089Q-S1098Q** | **S1403Q** | **S1431Q** | **S1542Q** | **S1739Q** | **S1873Q** | **S2189Q** | **S2439Q** |
| Mouse | KAHIS**SQ**NSELS | ESTEN**SQ**LNVKI | NNLDA**SQ**DTLFSAQF**SQ**EESME | IVNED**SQ**AAALA | DSCSD**SQ**ERESG | QTRRA**SQ**GLISA | CCGEK**SQ**S--QEKS | ECKDI**SQ**KQLSE | RGLKR**S**-**Q**EDEI- | YSYTG**SQ**LFEMH |
| Human | KTHIS**SQ**NSELS | DGTEN**SQ**LNVKI | NNLDV**SQ**DTLF-TQY**SQ**EEPME | MVNED**SQ**VQITP | ESTTE**SQ**DKENS | QTRRA**SQ**GLLSS | CCGEK**SQ**P--QEKS | DSKNV**SQ**E--SL | RGLKR**S**-**Q**EDEIS | HNYSG**SQ**LFEMH |
| Chimpanzee | KTHIS**SQ**NSELS | DGTEN**SQ**LNVKI | NNLDV**SQ**DTLF-TQY**SQ**EEPME | MVNED**SQ**VQITP | ESTTE**SQ**DKENN | QTRRA**SQ**GLLSS | CCGEK**SQ**P--QEKS | DSKNV**SQ**E--SL | RGLKR**S**-**Q**EDEIS | HNYSG**SQ**LFEMH |
| Marmoset | KTHIS**SQ**NSELS | DGAEN**SQ**FNVKI | NNLDV**SQ**DTLF-TQY**SQ**EESME | MVNED**SQ**SQITA | ESTTD**SQ**DKENN | QTRRA**SQ**GLLSS | CCGEKPQP--QEK- | DLENA**SQ**E--SL | RGLKR**S**-**Q**EDEIS | HNYSG**SQ**LFEMH |
| Rabbit | RTHISSPNLELS | DATEN**SQ**LNLKI | NNLDV**SQ**DTFLSSQY**SQ**EESME | MVNED**SQ**VQITP | ESTTD**SQ**DKENN | QTRRA**SQ**GLLSS | CCGEK**SQ**S--QEKS | DPKNIQQECSPF | RGLKRP-QEDENS | HNYSGNQLFEMH |
| Cow | ------------ | DMTEN**SQ**LNMKI | NNLDV**SQ**DTLF-SQY**SQ**EESME | TVNEDSHIQVTP | EPTTD**SQ**DKENN | QTRRA**SQ**GLLSS | CCGEK**SQ**S--QEKS | VSKDISLKCFSL | RGLKRP-QEDEIS | YNYSG**SQ**LFEMH |
| Horse | KTHIS**SQ**NSELS | EGREN**SQ**LNMKI | NNLDV**SQ**DALY-SQYGQEESME | MINEDSHVQITP | EPTTE**SQ**DKENN | QTRRA**SQ**GLLSS | CCGEK**SQ**P--QEKS | DSKDISLECSTF | RGLKRPLQEDEIS | HNYSG**SQ**LFEMH |
| Elephant | KTHIS**SQ**NPELS | DATES**SQ**LNVKI | NNLDV**SQ**DTLF-SQY**SQ**EESME | MVNED**SQ**VPLTP | ESIP-DQDKENN | QTRRA**SQ**GLLSS | CCGEKAQP--QEKP | DSKEEV**SQ**KNSL | RGQKRP-QEDEIP | HNYSG**SQ**LFEMH |
| Opossum | KTHIS**SQ**NSELN | EGTEN**SQ**LDAKI | NNLDI**SQ**DSFS--YH**TQ**EETME | QIPNDDSHIQVS | EGTPDRQDKENG | QTRRASLGLLSS | CCGEKSKL--REKF | SINN**SQ**---DSF | RGIKRQ-QEDEAS | HNYSG**SQ**LFEMQ |
| Platypus | QVHISHQNSELN | EPTEN**TQ**LEAKI | NNLDV**SQ**DTALFSQY**SQ**EESME | MSNED**SQ**GPQIA | ECATG**SQ**DKENS | QTRRA**SQ**GLLSS | CCGEK**SQ**S--QEKS | NDDDSL---DPL | RGIKRP-QEDETP | HQYSG**SQ**LFEMQ |
| Chicken | KAHISSENSELS | DEAEN**SQ**WDAKL | NNLDS**SQ**DTTLFSQY**TQ**SQEDS | VMGDG**SQ**GPHAS | EMAAG**SQ**DKEDG | HTRRS**SQ**GLLSS | CCSKRVKQ--QTS- | ------------ | RGVKRH-HEDDSL | RSYPGSRLFEMQ |
| Lizard | ------------ | DSVSIRVWHWLY | ---------------------- | TISNVGQSPQVS | EGASGSPDKENS | QTRRT**SQ**GLLIS | SSPEKS-S--ETKG | ------------ | RGVKRQ-KEEDSP | LNYSVSELFEMQ |
| Xenopus | KAHISNQNSDLS | ENMDN**SQ**LEAKI | NNLDA**SQ**DTTLFTQY**SQ**SQDNS | ANNDT**SQ**IQQAT | DGVKSIADKGQK | QTRRS**SQ**GLVLA | CCGKASNN--KEST | ETKTVAAPEETM | KGVKRQ-QENDSP | CKYSA**SQ**LFDMQ |
| Zebrafish | LVHISSENEELC | YMSES**SQ**LEPQI | NNLDASMDTTVFSQY**TQ**SQEES | ANKDL**SQ**TELSP | TQLEESTEKETN | RTRRS-RGLLS- | CFSNNRDVFSQDSD | ------------ | KGQKRA-CEEETP | SQHSA**SQ**LGLMH |
| Shark | KGHISSSNSEVG | KYSEH**SQ**IDTKI | NNLDV**SQ**DSHLFSQY**TQ**SQDSS | QDEQNEQTPNTP | TGQAG**SQ**RKKKG | QTRRS**SQ**GISQV | CCKGSTKQ--KRKS | KV-ATSAPCDKT | KGIKRP-SENESP | NEYSG**SQ**LFTMH |
| Drosophila | ------------ | ------------ | NDMSQ**SQ**DSASIKPWTPKKVVI | GTDHTSTPIQAP | KLRCESLDDVTL | ------------ | SCRNGLLA--QVPE | ------------ | ------------- | ------------ |
| Fission yeast | LSDRCSNNSEGS | NHQSSEKMSDIL | KNKRQKGDVKKIDETK-NEATD | ------------ | ------------ | ------------ | -------------- | ------------ | ------------- | ------------ |
| Baker's yeast | ------------ | ------------ | GMKEPPSSIQISSQISAKDSDS | NINDDFVPVEEN | MQGSK**SQ**IKEKL | FSKK-SRRLVAR | -------------- | ------------ | ------------- | ------------ |

^a^ The alignment was performed simultaneously on full-length RIF1 proteins from all 18 species using the multiple sequence alignment program Clustal Omega (clustalo version 1.2.4). Five amino acid residues on either side of the SQ/TQ motifs are included for each peptide.

^b^ SQ motifs (or equivalent TQ motifs at the same position) conserved in the analysed species are highlighted in bold. Underlined amino acid residues indicate either a TQ replacing the SQ motif at the same position or an offset SQ site (-/+ 1 position based on alignment output). The “–“ symbol indicates complete lack of or only partially limited alignment of the corresponding RIF1 protein in that region.

^c^ The alignments of peptides containing T1450Q, T1688Q, T1893Q, T2015Q, and S2211Q from mouse RIF1 are not included in the table because these SQ/TQ motifs are not conserved in human RIF1. The S1715Q motif is included in the alignment of peptides containing S1713Q because of proximity but the motif is not highlighted because it is not conserved in human RIF1.

^d^ The alignments of peptides containing T956Q, T1095Q, T1518Q, S1777Q, S1851Q, S2117Q, S2124Q, and T2246Q from human RIF1 are not included in the table because these motifs are not conserved in mouse RIF1.

Cells with thicker borders denote the SQ motifs investigated in this study.

﻿
