# Supplementary figures and images for: Protection of nascent DNA at stalled replication forks is mediated by phosphorylation of RIF1 intrinsically disordered region
Source: eLife. 2022 Apr 13;11:e75047. doi: 10.7554/eLife.75047 (PMC9007588; doi:10.7554/eLife.75047)

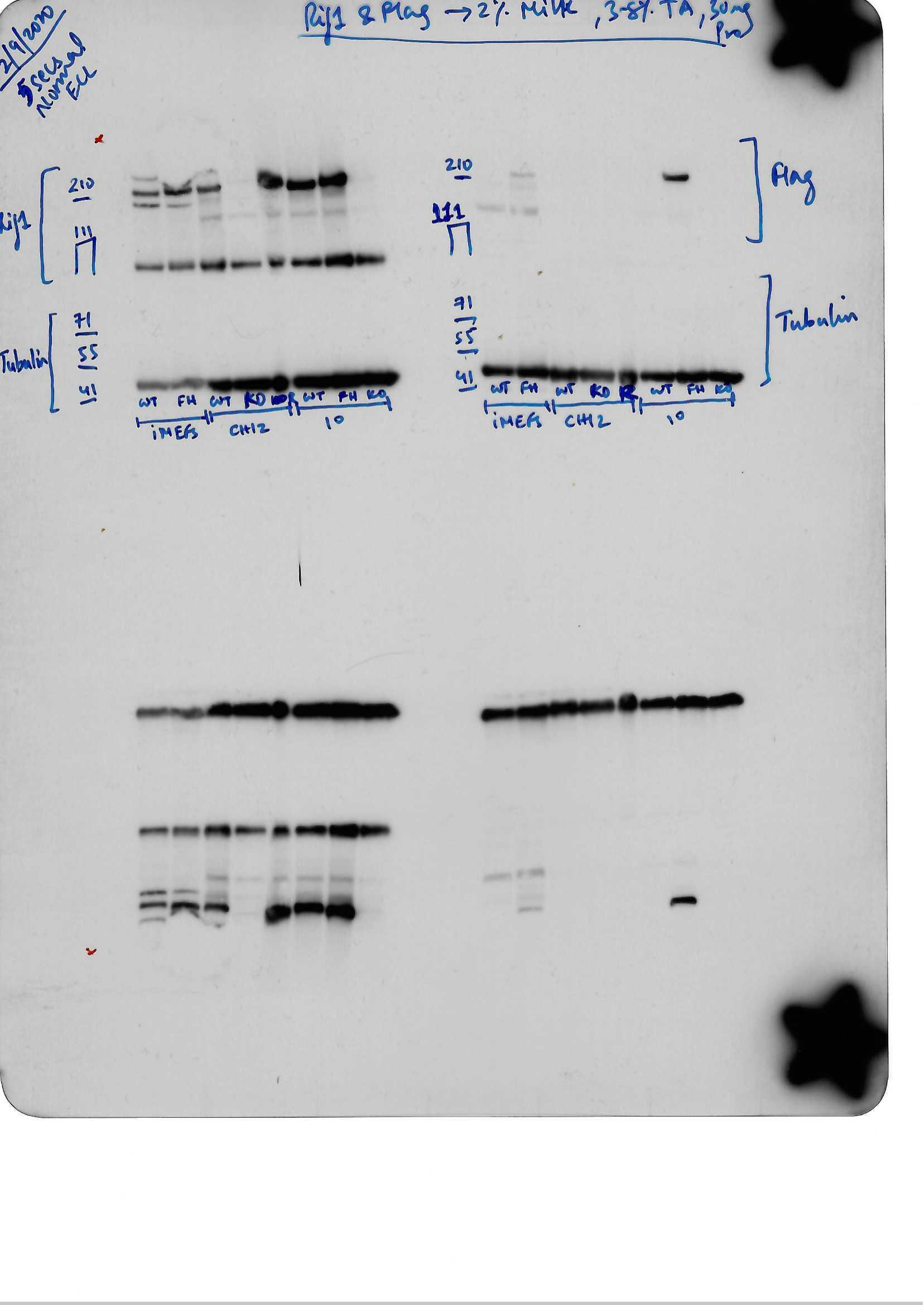

Supplement: Figure 1—source data 3. [file elife-75047-fig1-data3.zip › 75047Figure1SourceData3.tiff]

**C**

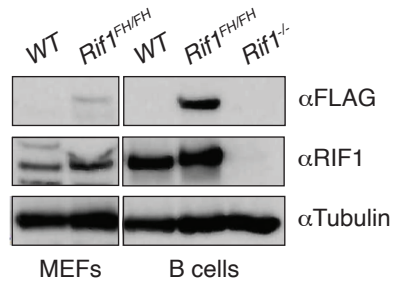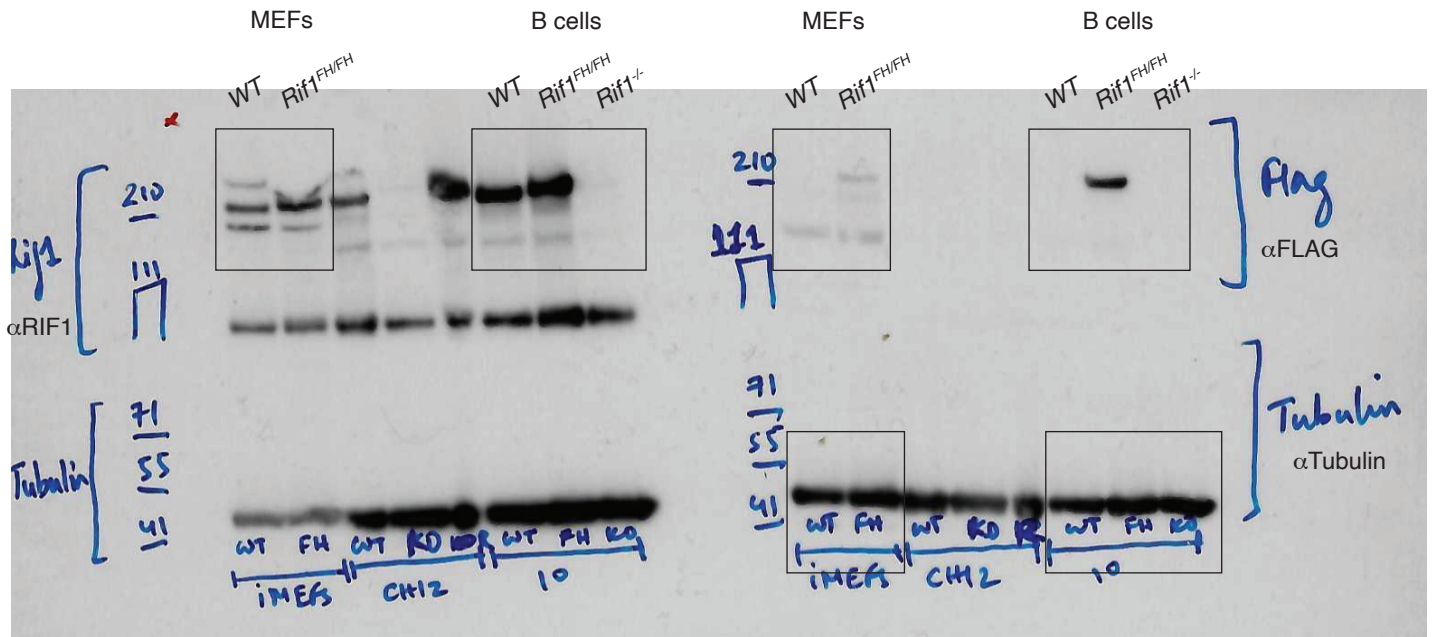

**Figure 1**

Supplement: Figure 1—source data 4. [file elife-75047-fig1-data4.zip › 75047Figure1SourceData4.pdf]

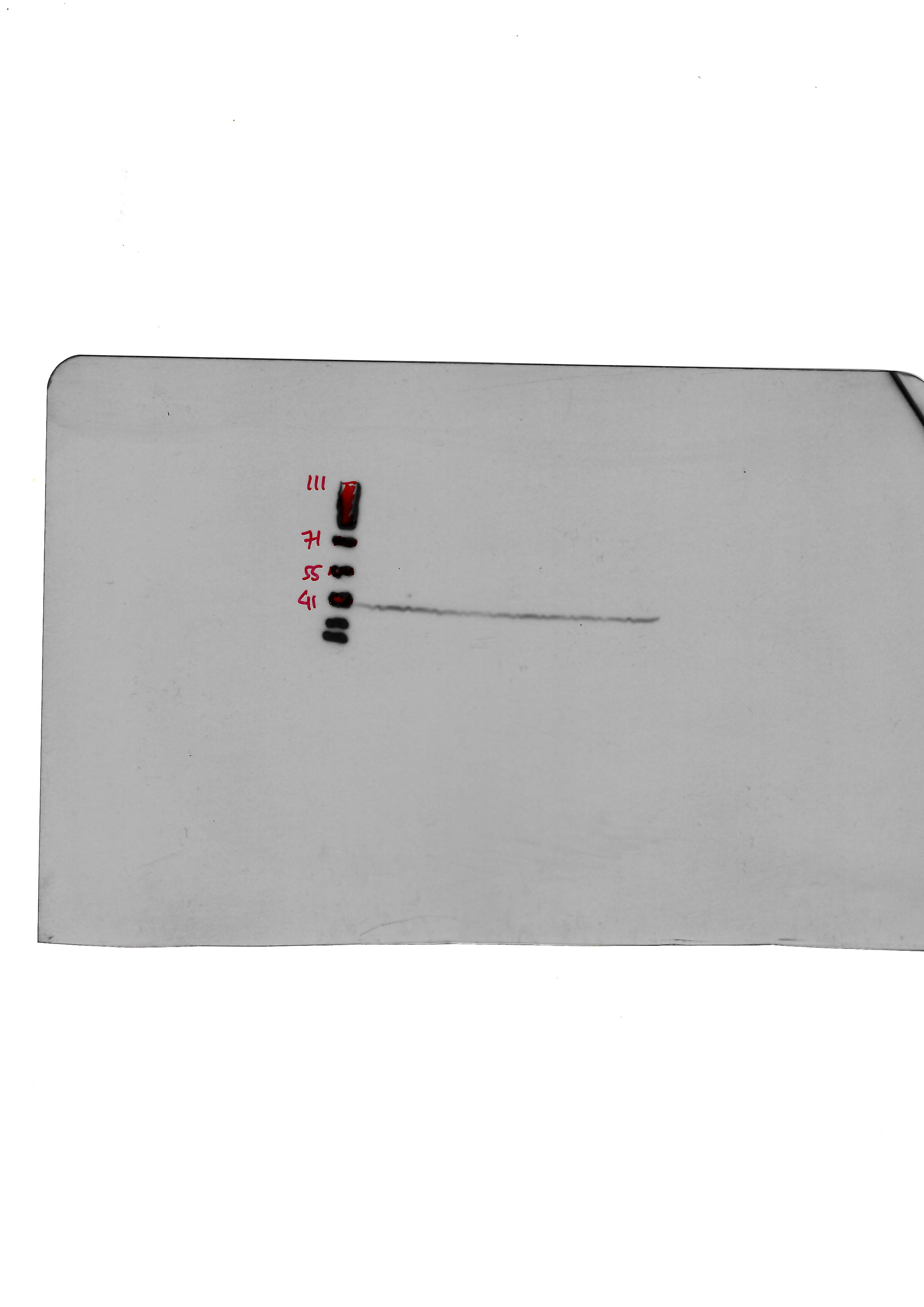

Supplement: Figure 2—source data 2. [file elife-75047-fig2-data2.zip › 75047Figure2SourceData2.tiff]

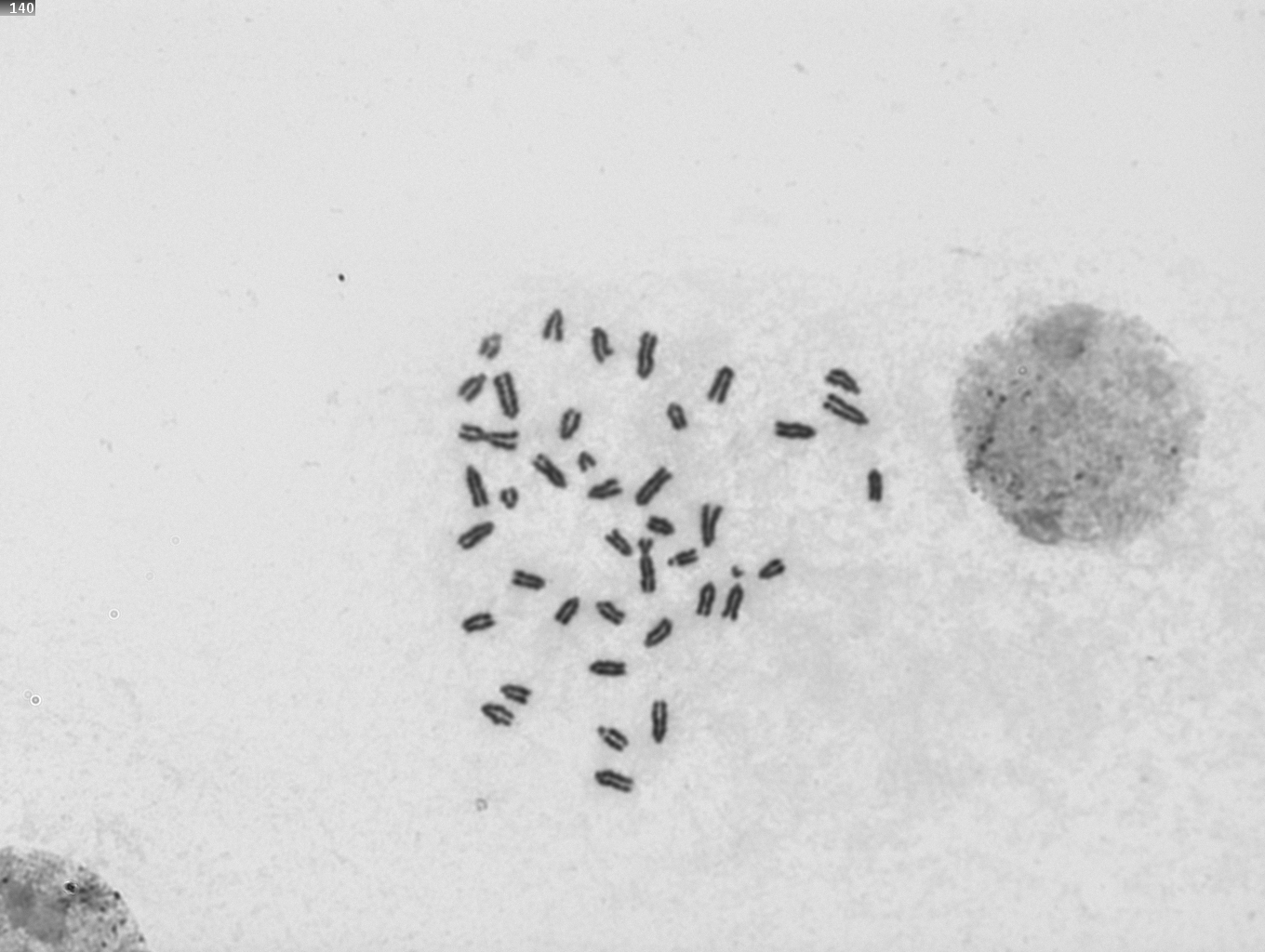

Supplement: Figure 2—figure supplement 1—source data 1. [file elife-75047-fig2-figsupp1-data1.zip › 75047Figure2S1SourceData1.TIF]

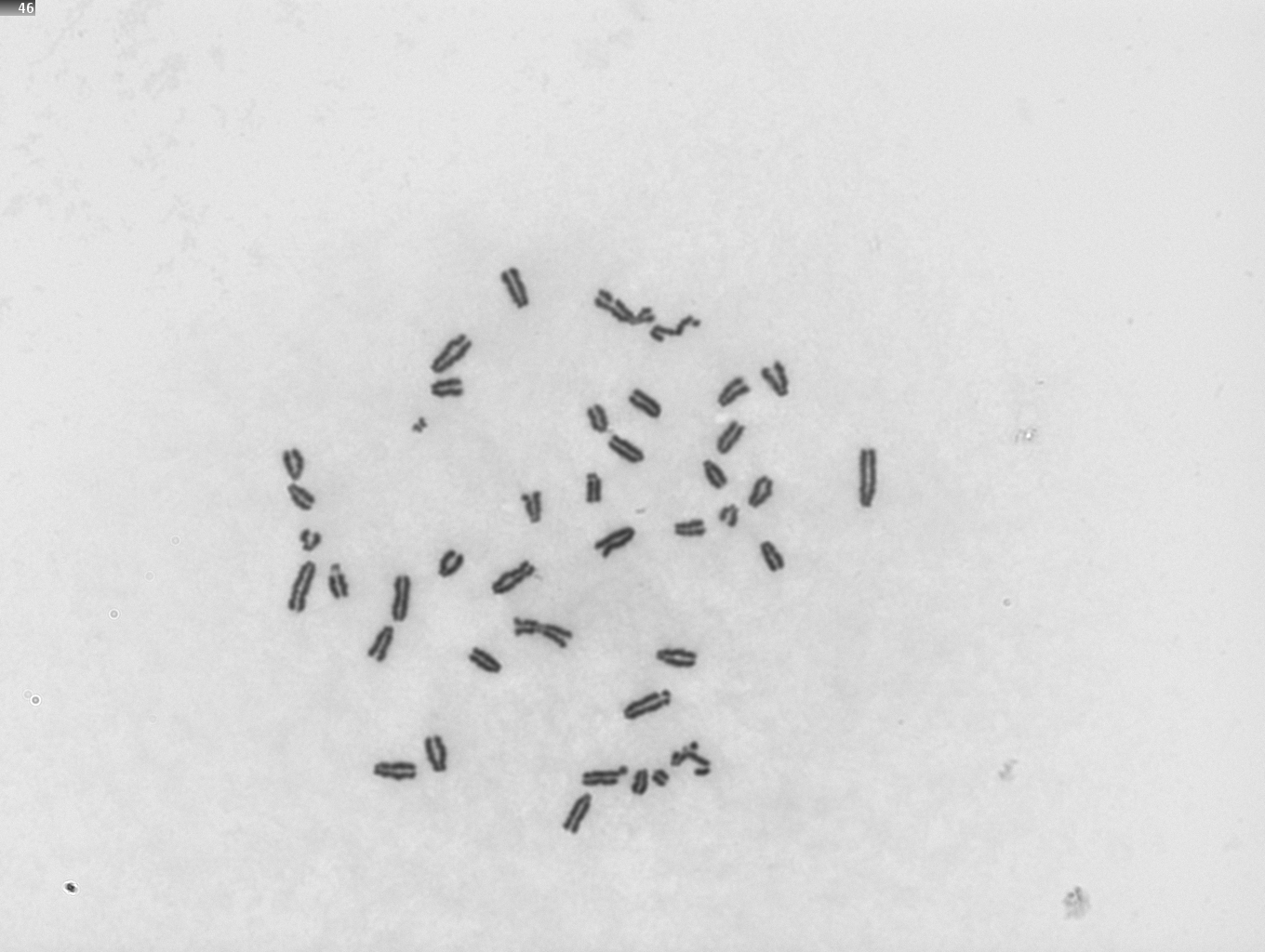

Supplement: Figure 2—figure supplement 1—source data 2. [file elife-75047-fig2-figsupp1-data2.zip › 75047Figure2S1SourceData2.TIF]

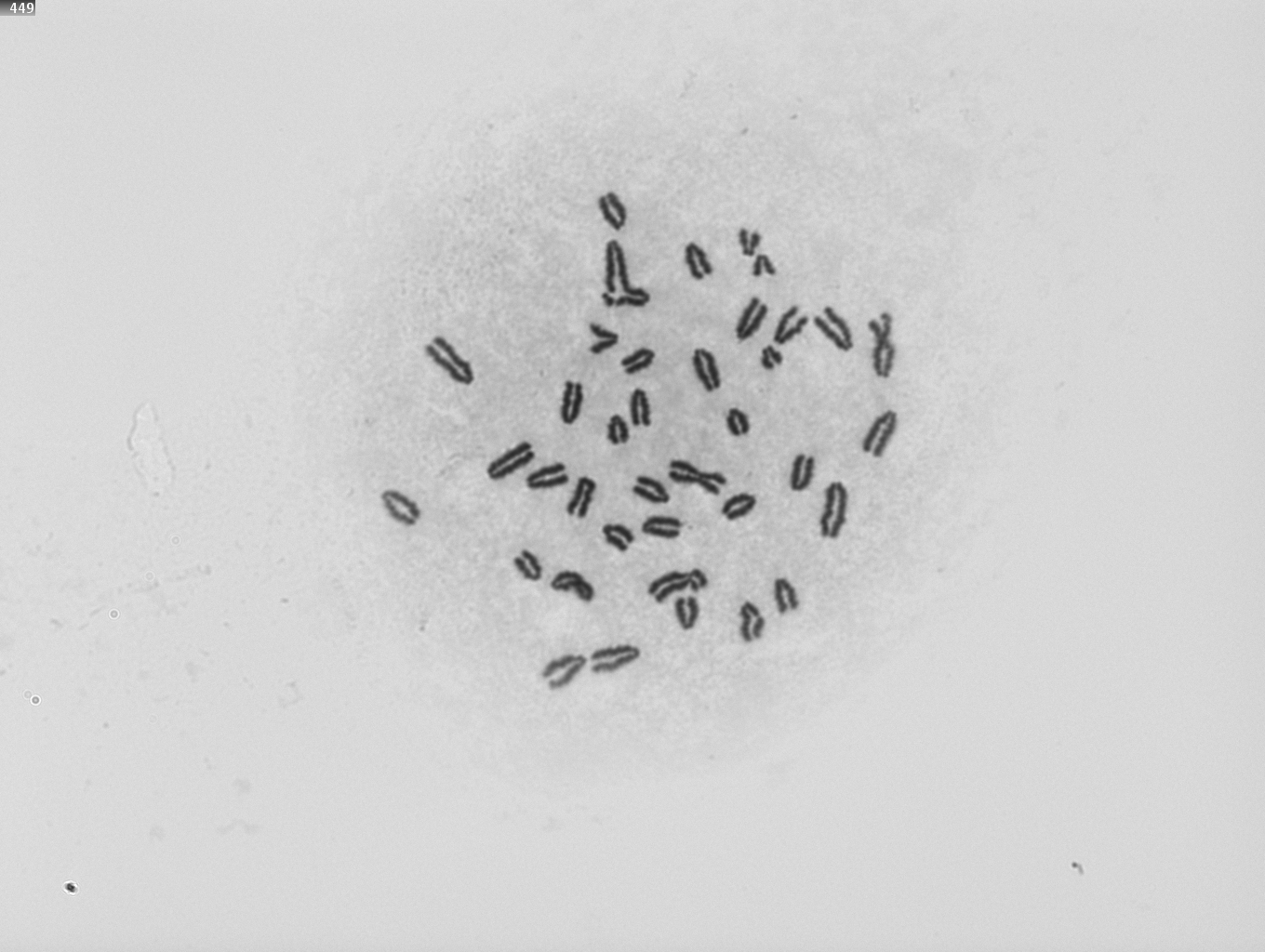

Supplement: Figure 2—figure supplement 1—source data 3. [file elife-75047-fig2-figsupp1-data3.zip › 75047Figure2S1SourceData3.TIF]

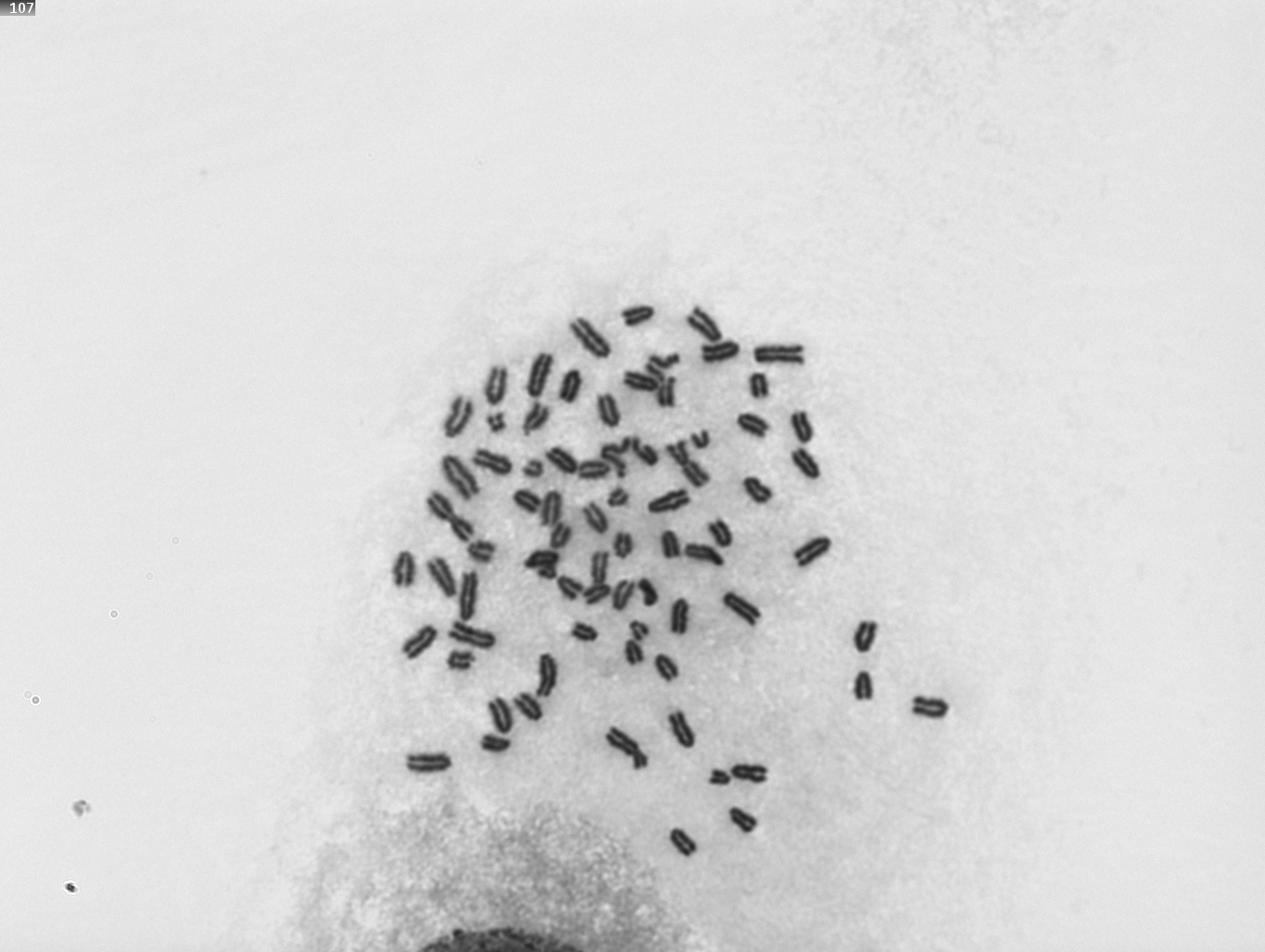

Supplement: Figure 2—figure supplement 1—source data 4. [file elife-75047-fig2-figsupp1-data4.zip › 75047Figure2S1SourceData4.TIF]

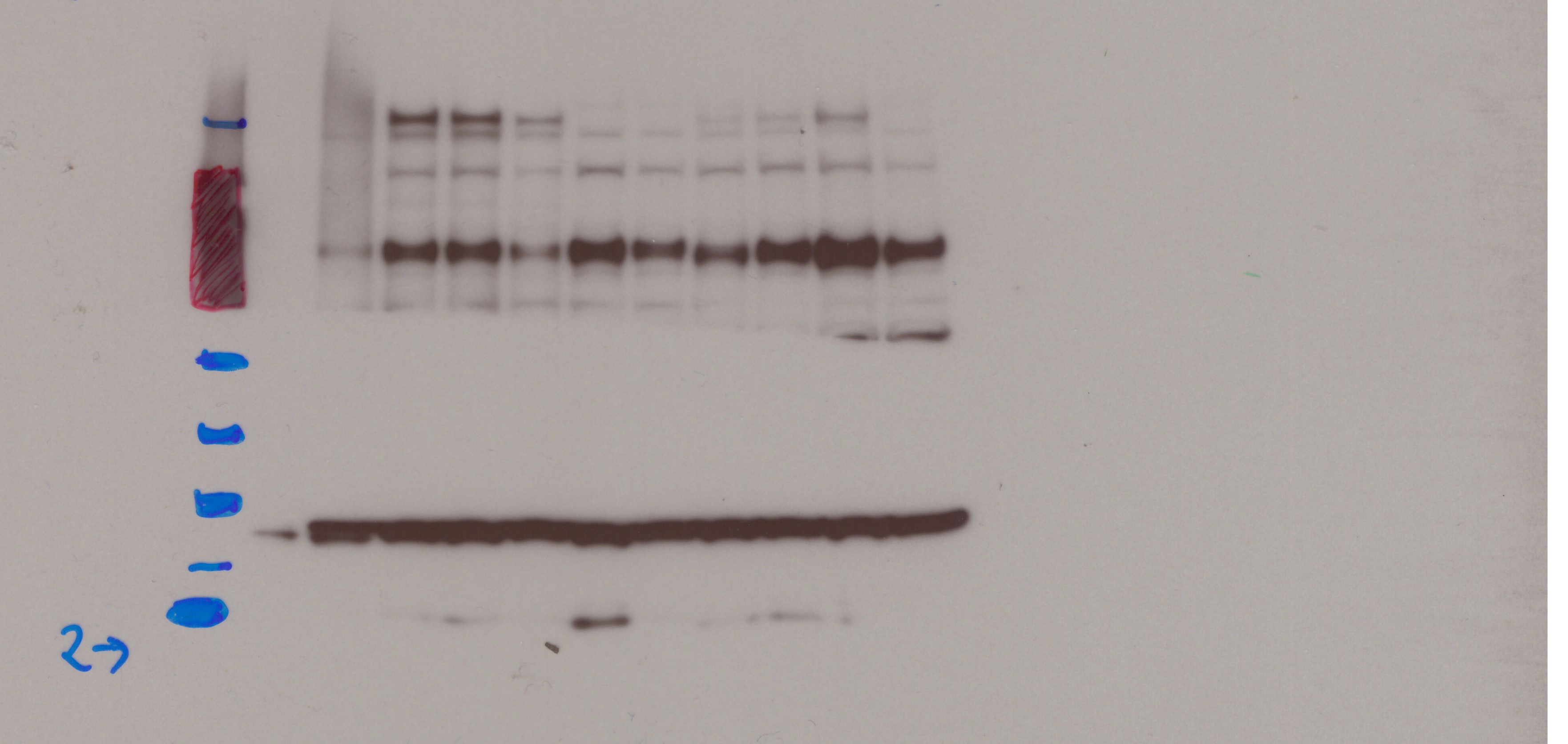

Supplement: Figure 2—figure supplement 2—source data 1. [file elife-75047-fig2-figsupp2-data1.zip › 75047Figure2S2SourceData1.jpg]

C

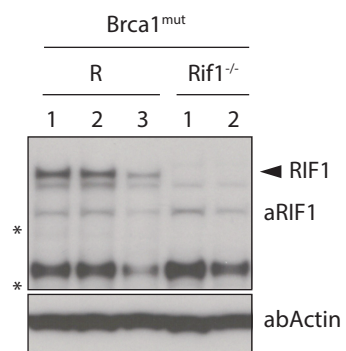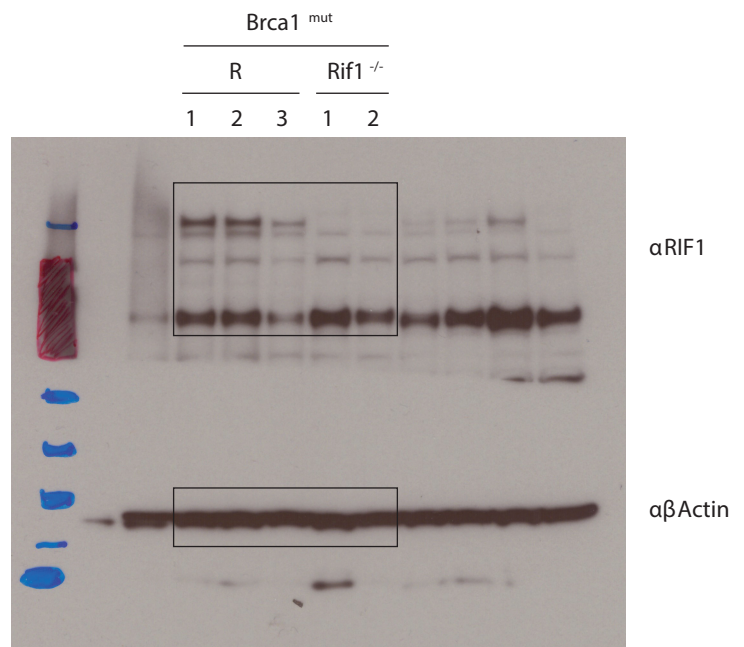

Figure 2 - figure supplement 2

Supplement: Figure 2—figure supplement 2—source data 2. [file elife-75047-fig2-figsupp2-data2.zip › 75047Figure2S2SourceData2.pdf]

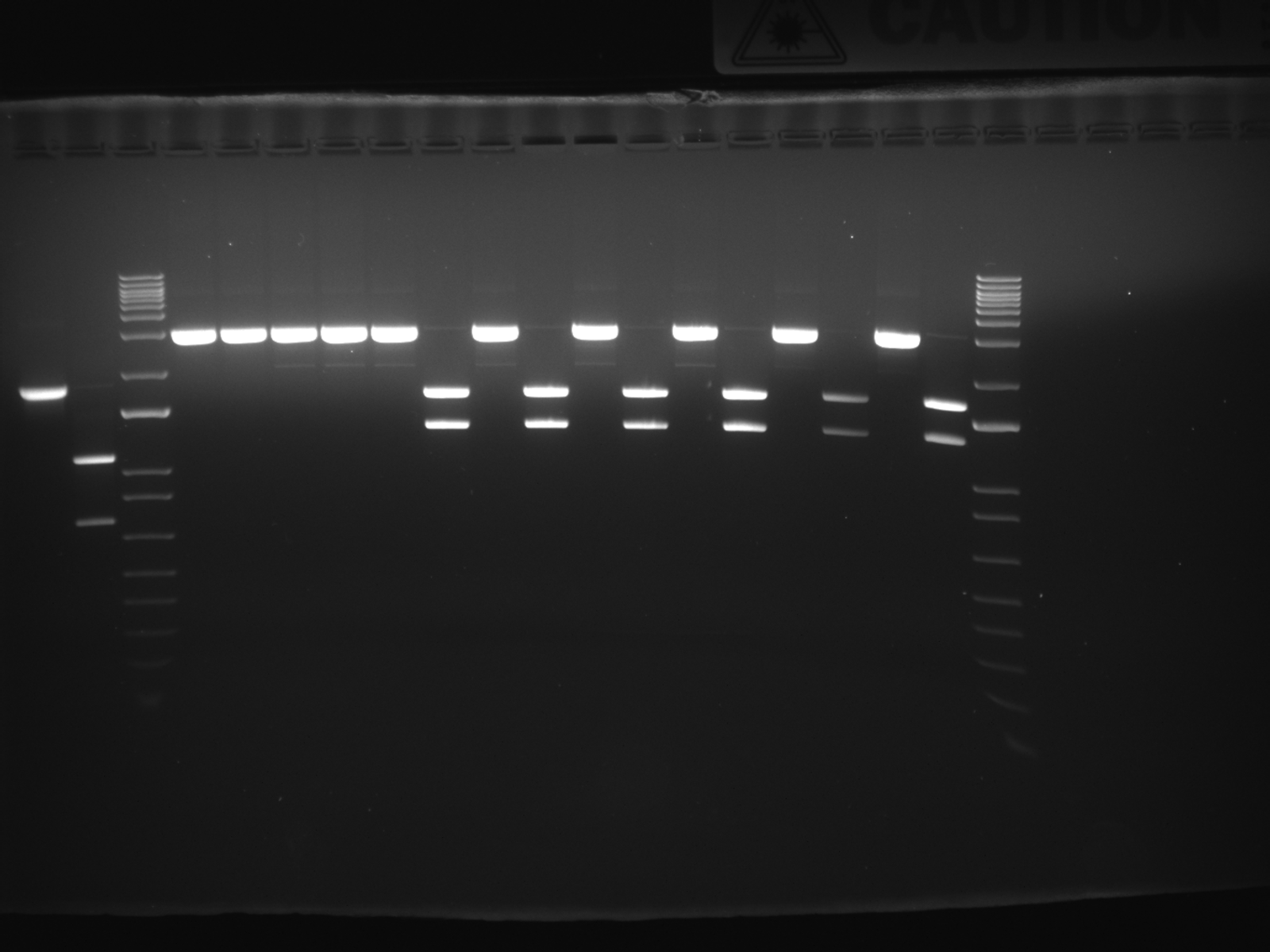

Supplement: Figure 2—figure supplement 2—source data 3. [file elife-75047-fig2-figsupp2-data3.zip › 75047Figure2S2SourceData3.Tif]

G

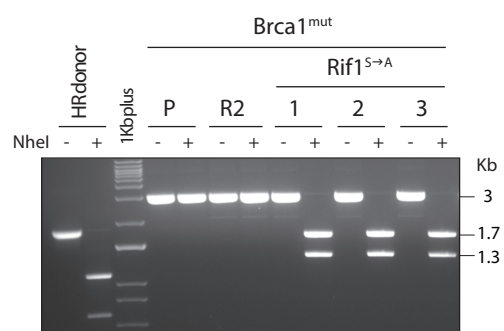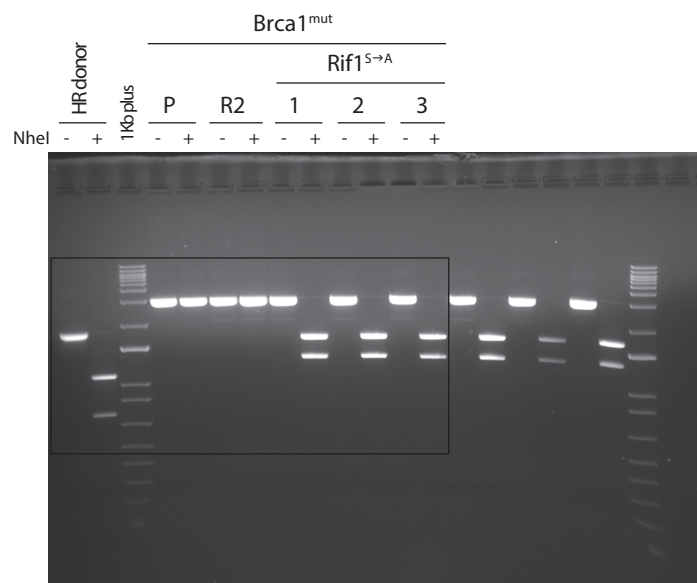

Figure 2 - figure supplement 2

Supplement: Figure 2—figure supplement 2—source data 4. [file elife-75047-fig2-figsupp2-data4.zip › 75047Figure2S2SourceData4.pdf]

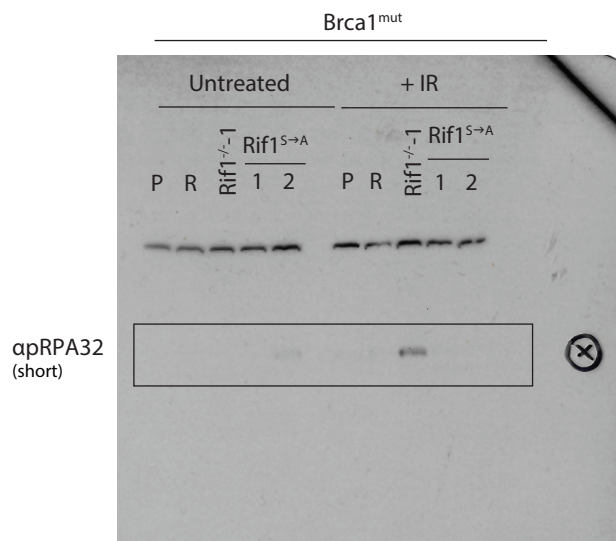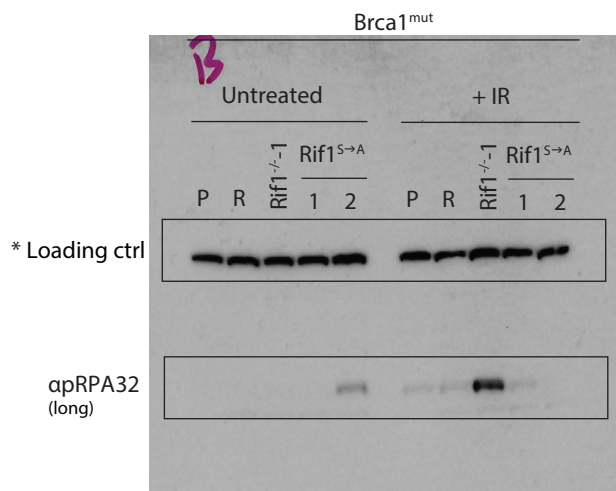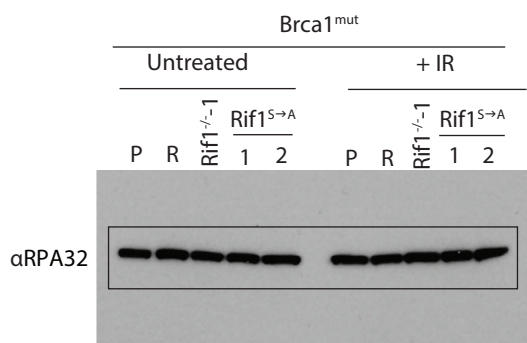

H

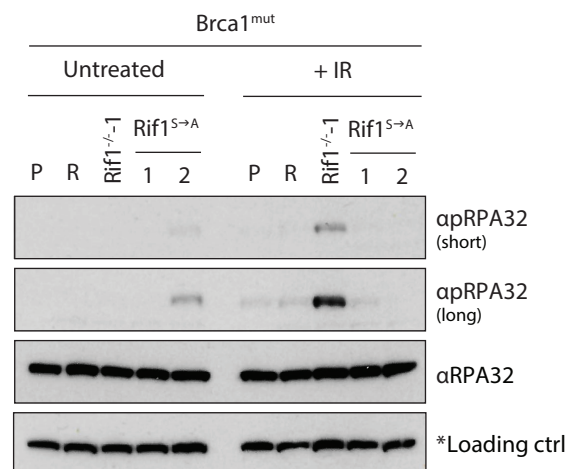

Figure 2 - figure supplement 2

Supplement: Figure 2—figure supplement 2—source data 8. [file elife-75047-fig2-figsupp2-data8.zip › 75047Figure2S2SourceData8.pdf]

A

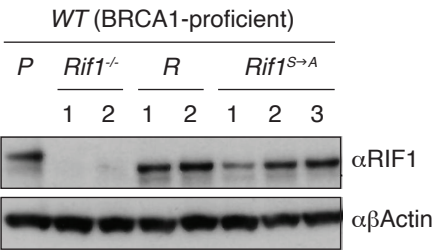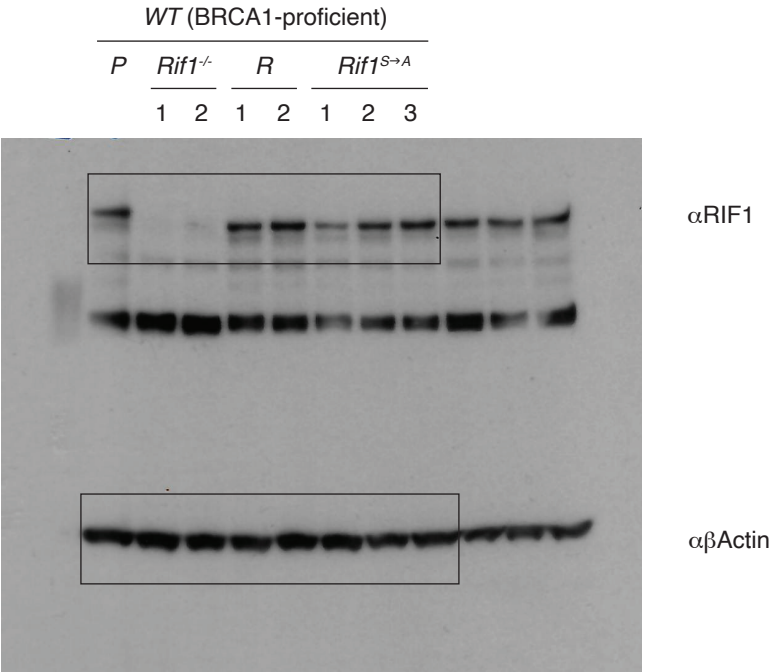

Figure 3

Supplement: Figure 3—source data 2. [file elife-75047-fig3-data2.zip › 75047Figure3SourceData2.pdf]

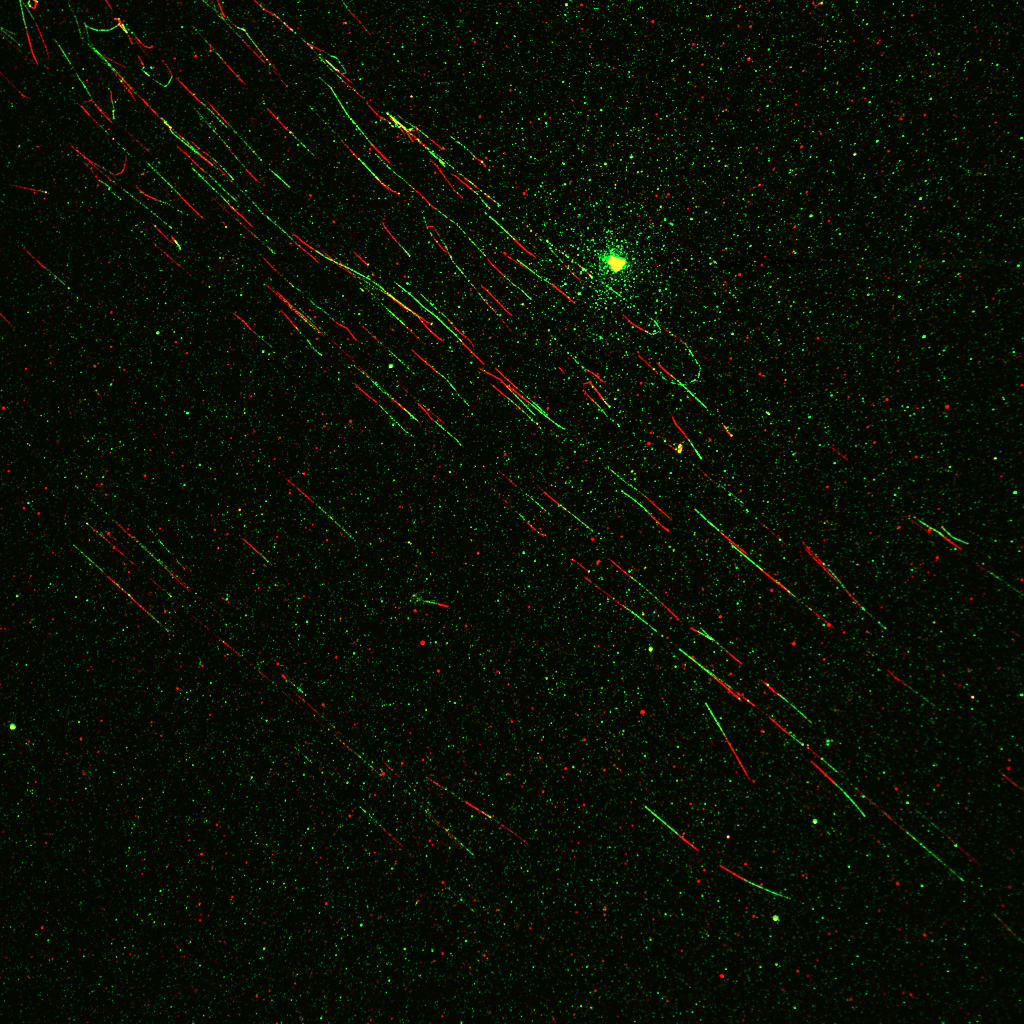

Supplement: Figure 3—source data 3. [file elife-75047-fig3-data3.zip › 75047Figure3SourceData3.tif]

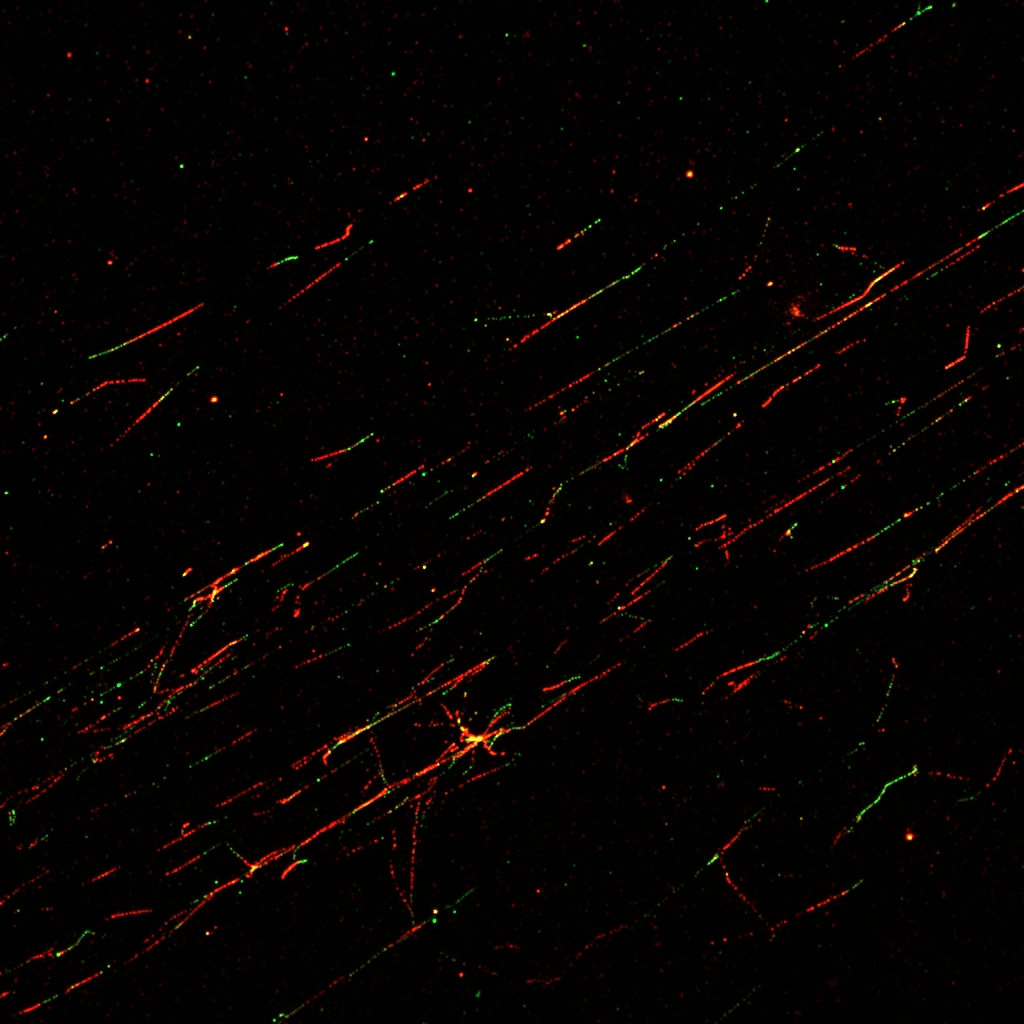

Supplement: Figure 3—source data 4. [file elife-75047-fig3-data4.zip › 75047Figure3SourceData4.tif]

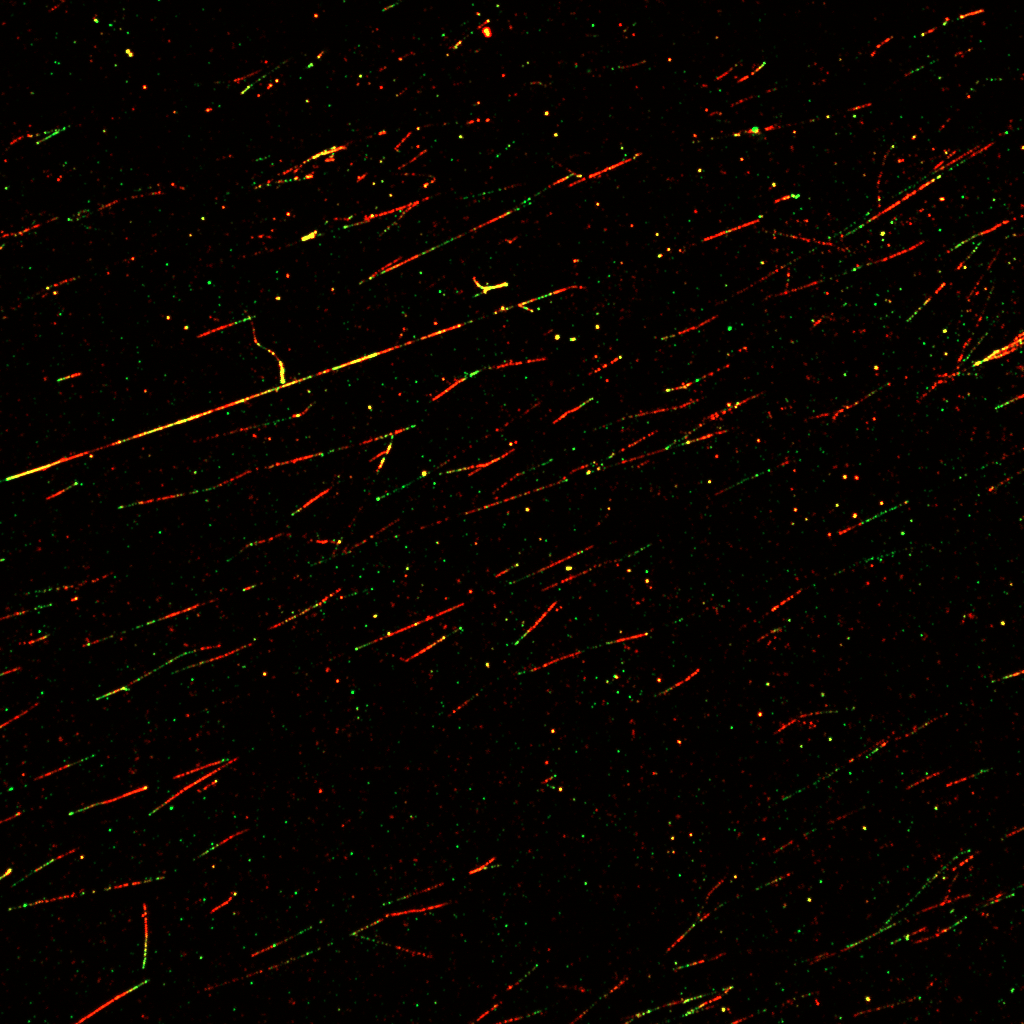

Supplement: Figure 3—source data 5. [file elife-75047-fig3-data5.zip › 75047Figure3SourceData5.tif]

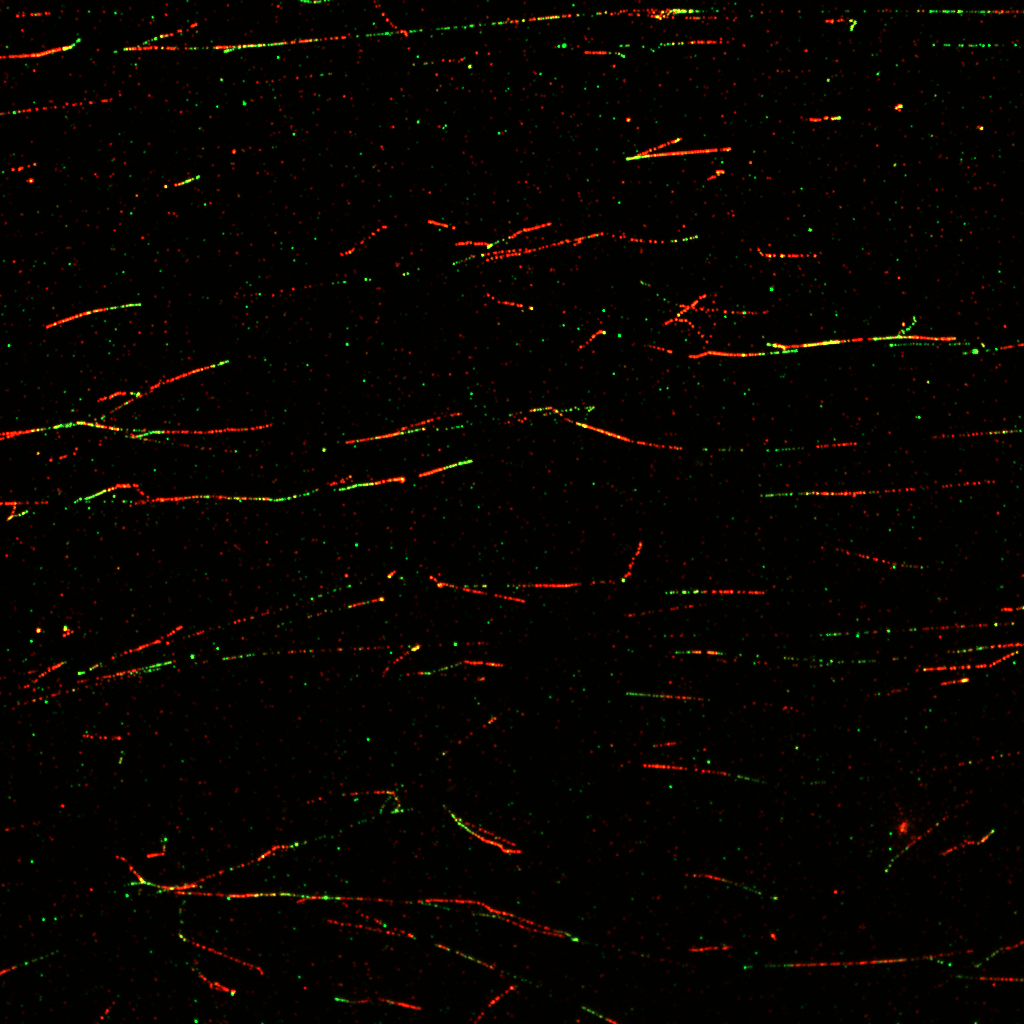

Supplement: Figure 3—source data 6. [file elife-75047-fig3-data6.zip › 75047Figure3SourceData6.tif]

B

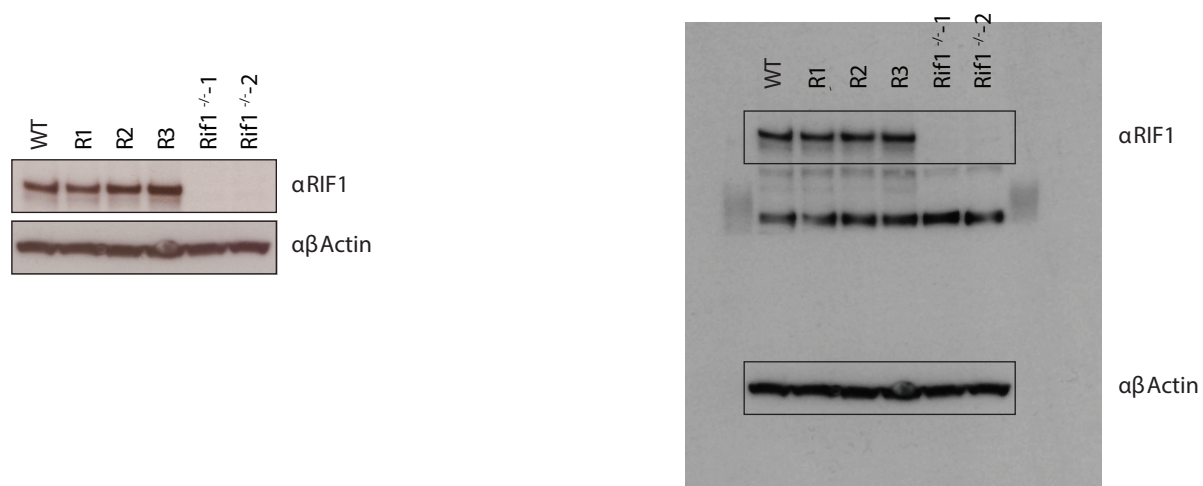

Figure 3 - figure supplement 1

Supplement: Figure 3—figure supplement 1—source data 2. [file elife-75047-fig3-figsupp1-data2.zip › 75047Figure3S1SourceData2.pdf]

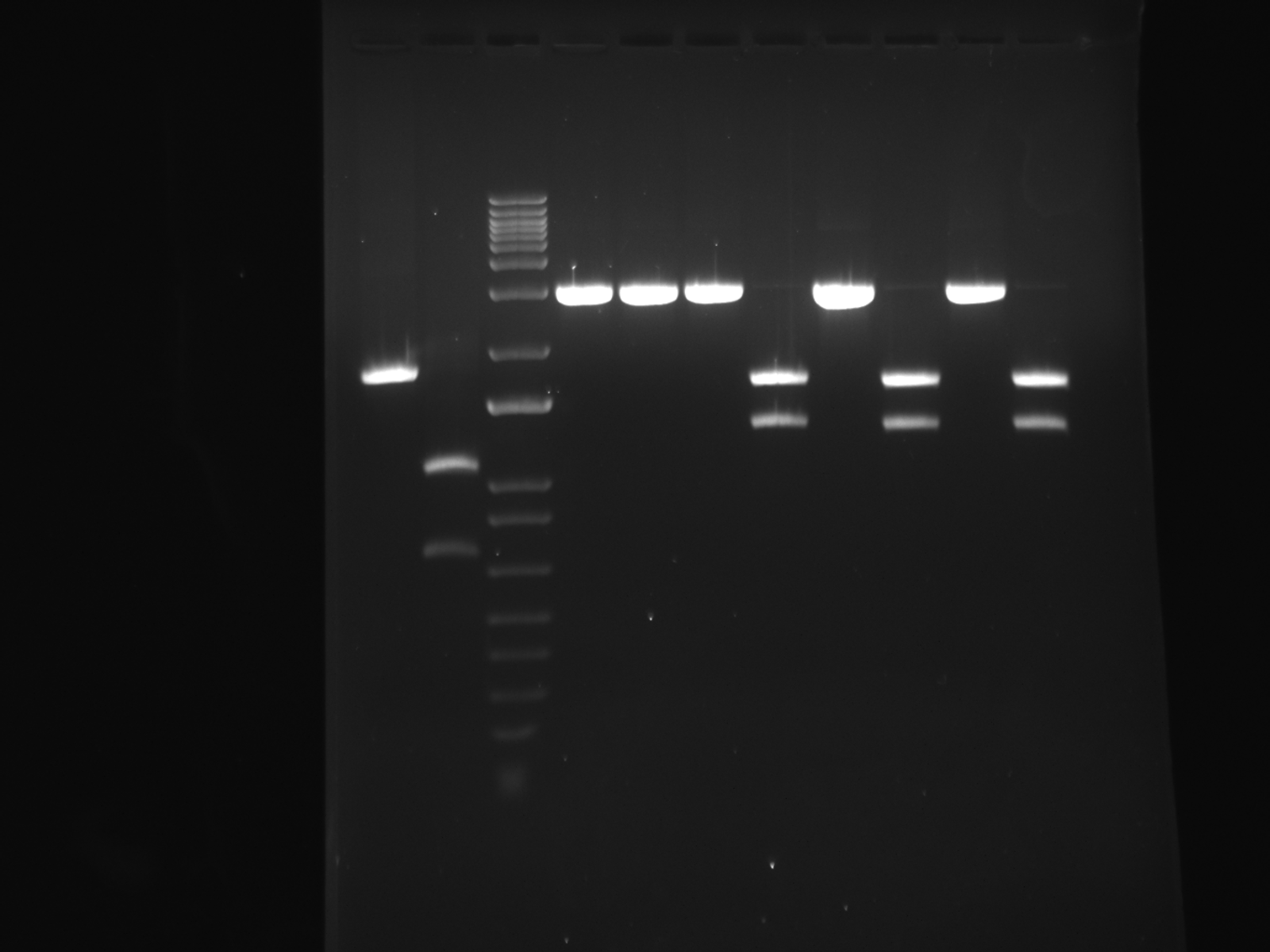

Supplement: Figure 3—figure supplement 1—source data 3. [file elife-75047-fig3-figsupp1-data3.zip › 75047Figure3S1SourceData3.Tif]

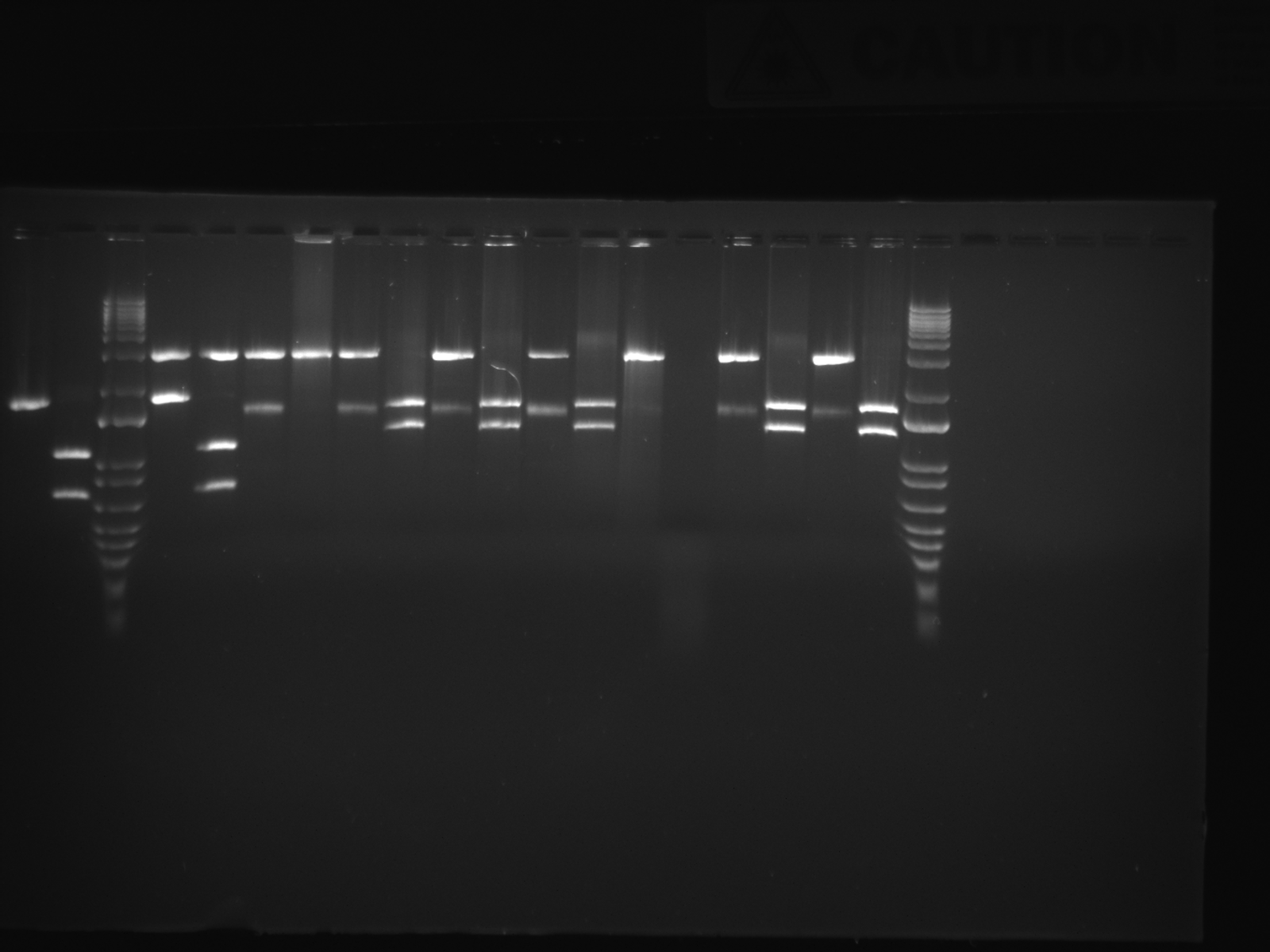

Supplement: Figure 3—figure supplement 1—source data 4. [file elife-75047-fig3-figsupp1-data4.zip › 75047Figure3S1SourceData4.Tif]

C

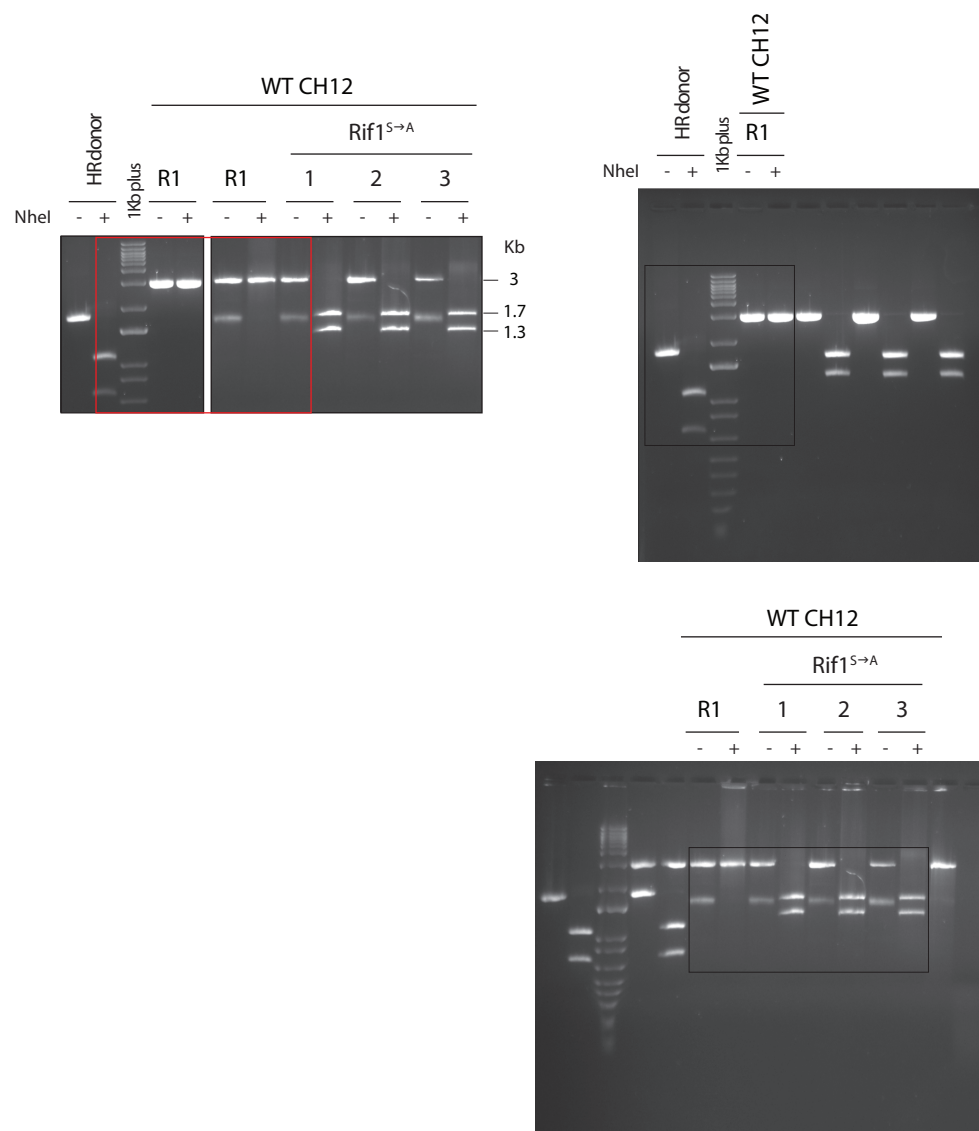

Figure 3 - figure supplement 1

Supplement: Figure 3—figure supplement 1—source data 5. [file elife-75047-fig3-figsupp1-data5.zip › 75047Figure3S1SourceData5.pdf.pdf]

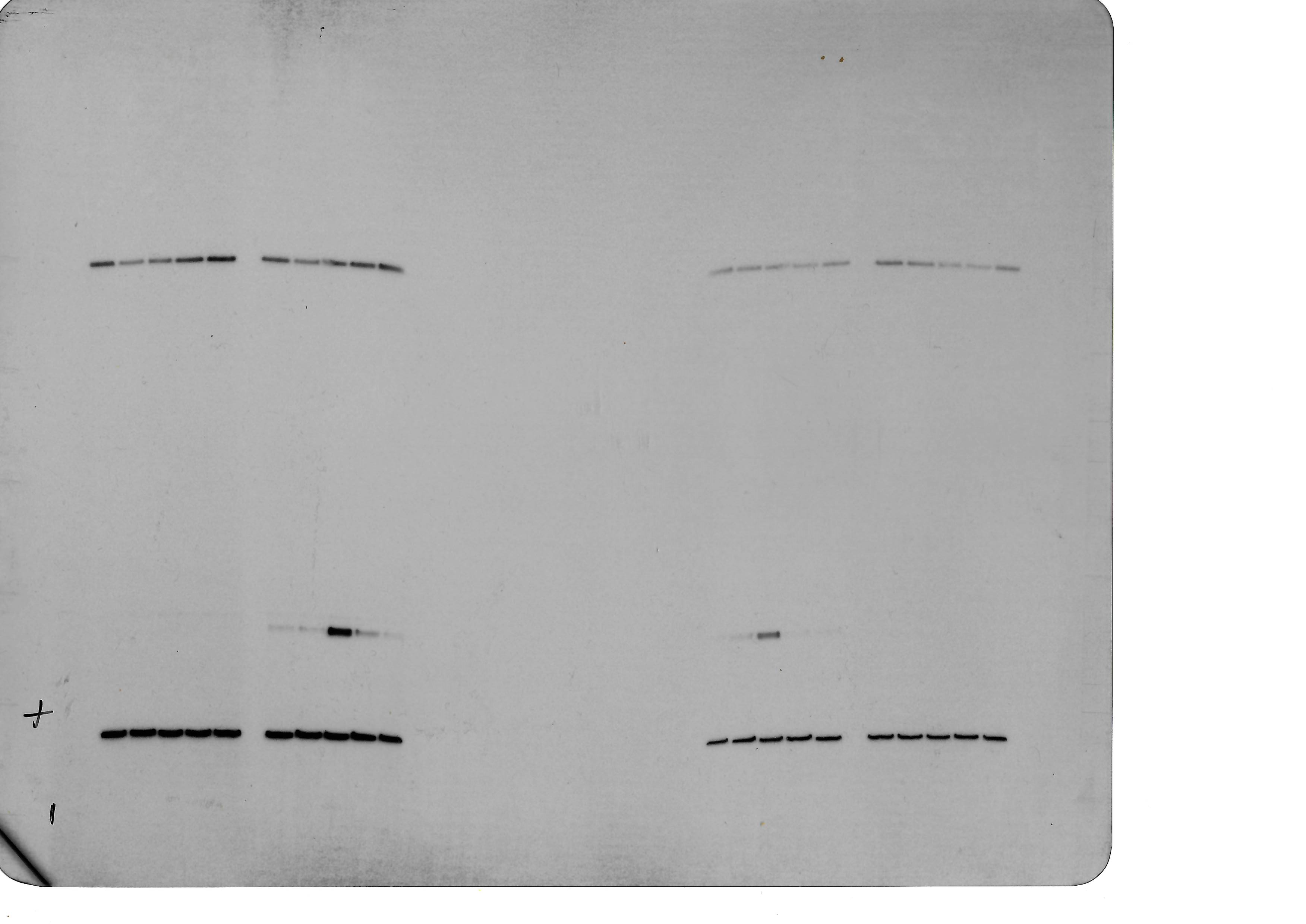

Supplement: Figure 3—figure supplement 1—source data 6. [file elife-75047-fig3-figsupp1-data6.zip › 75047Figure3S1SourceData6.tif]

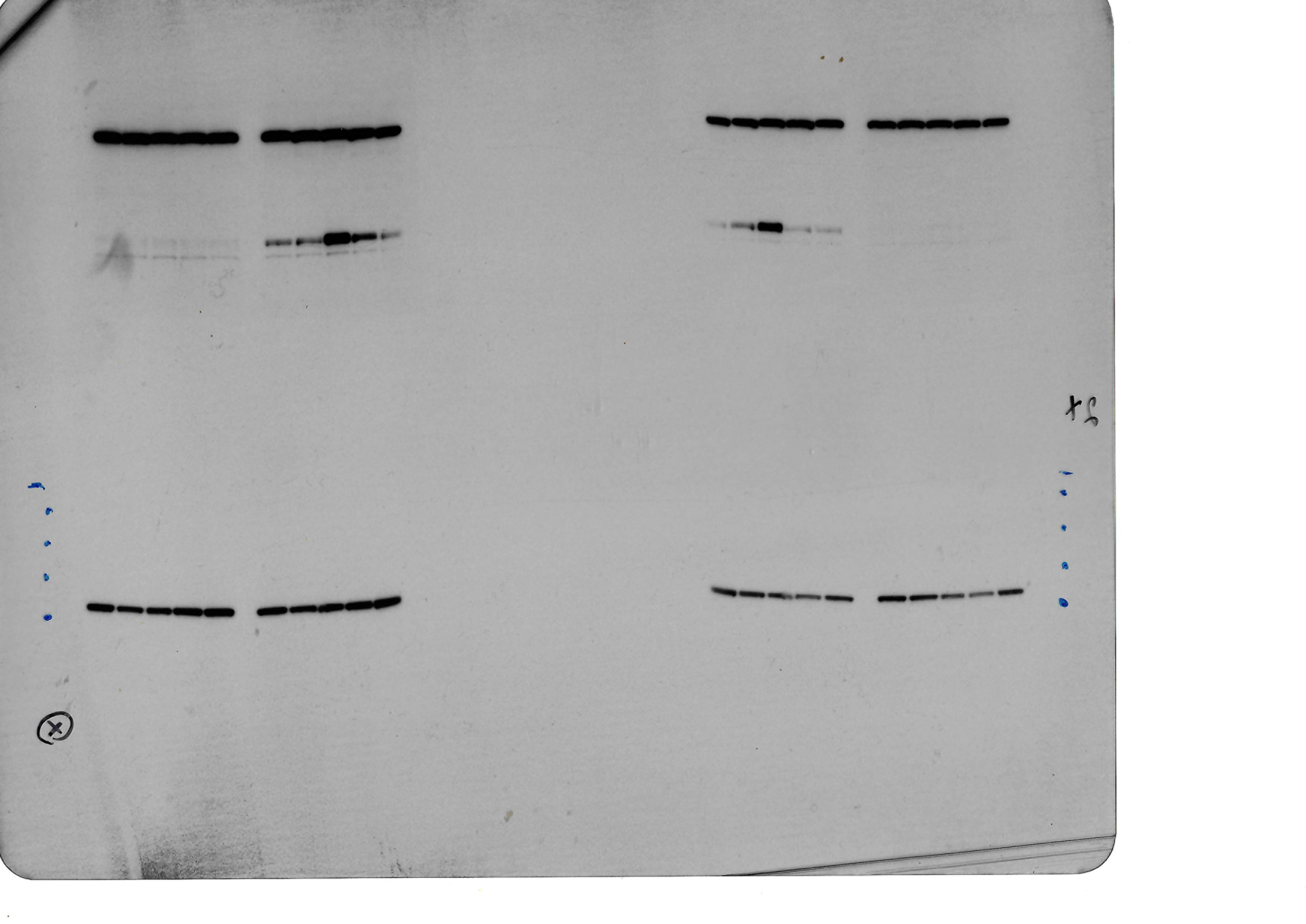

Supplement: Figure 3—figure supplement 1—source data 7. [file elife-75047-fig3-figsupp1-data7.zip › 75047Figure3S1SourceData7.tif]

E

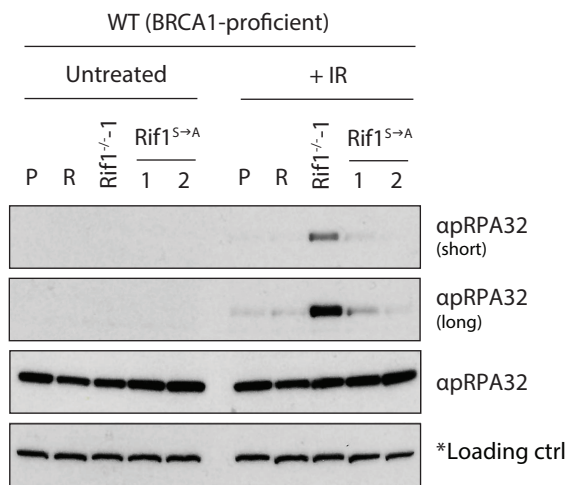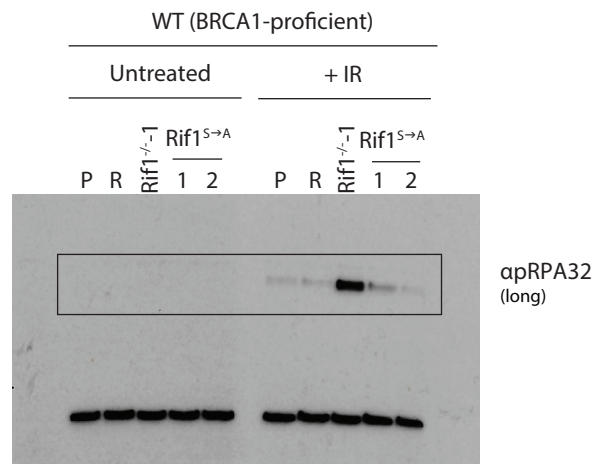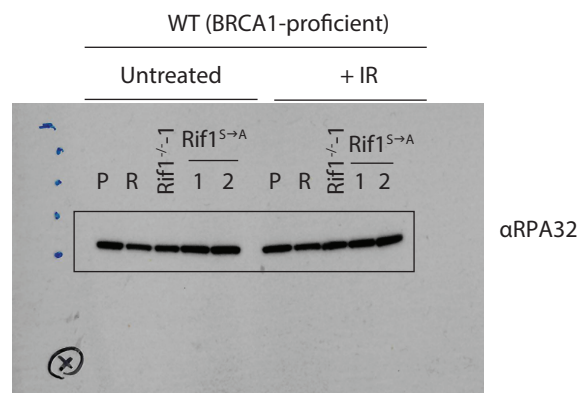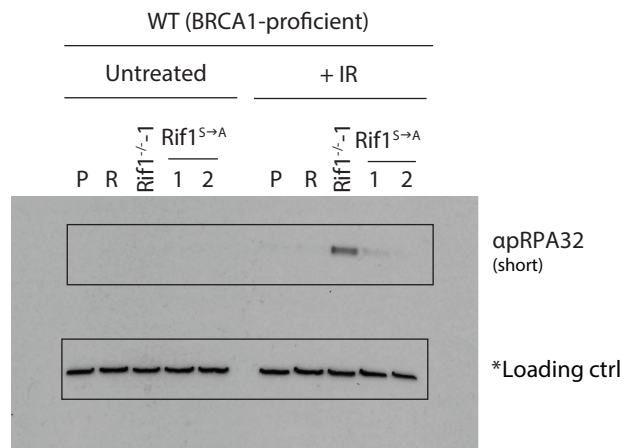

Figure 3 - figure supplement 1

Supplement: Figure 3—figure supplement 1—source data 8. [file elife-75047-fig3-figsupp1-data8.zip › 75047Figure3S1SourceData8.pdf]

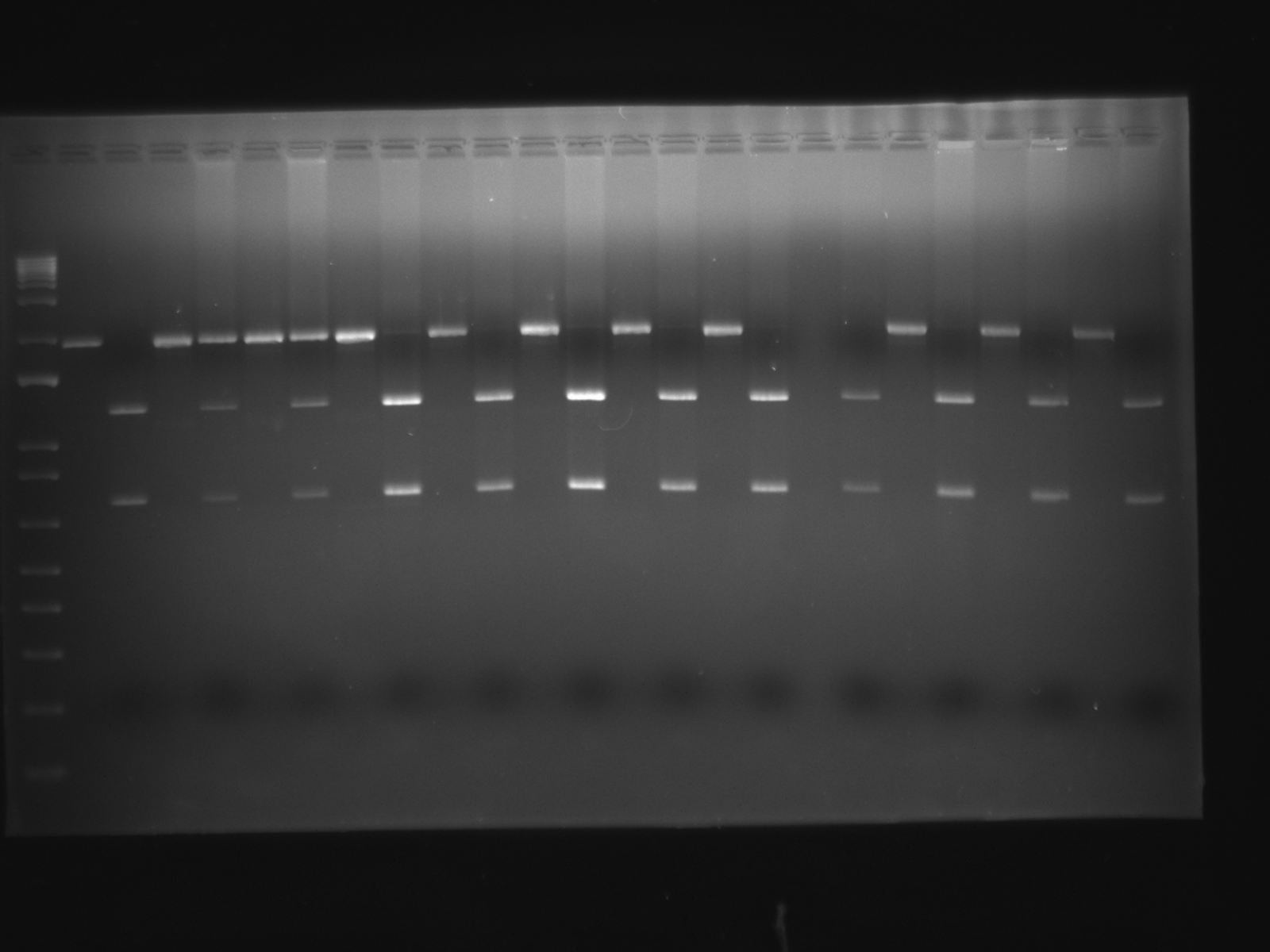

Supplement: Figure 3—figure supplement 1—source data 9. [file elife-75047-fig3-figsupp1-data9.zip › 75047Figure3S1SourceData9.Tif]

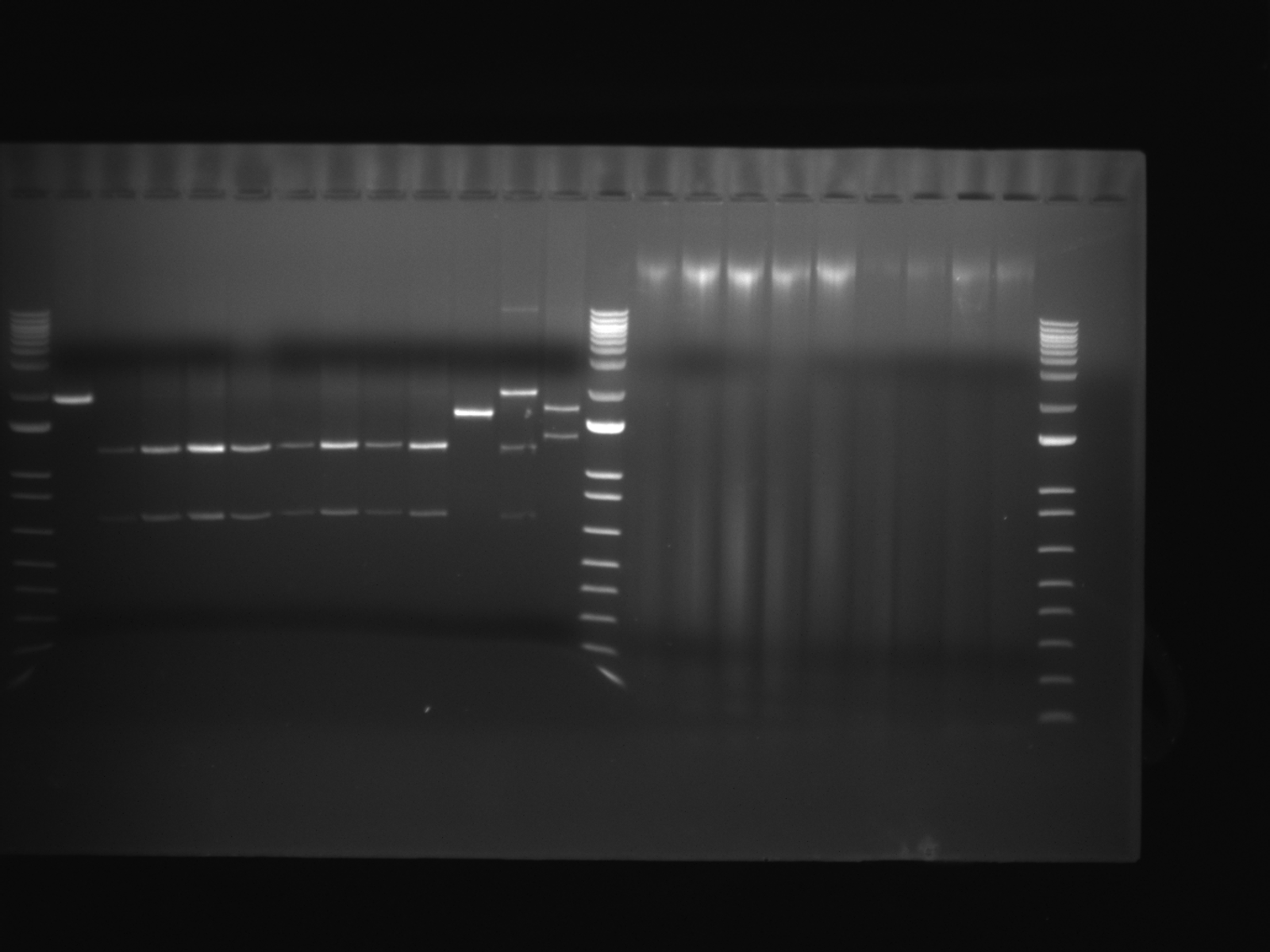

Supplement: Figure 3—figure supplement 1—source data 10. [file elife-75047-fig3-figsupp1-data10.zip › 75047Figure3S1SourceData10.Tif]

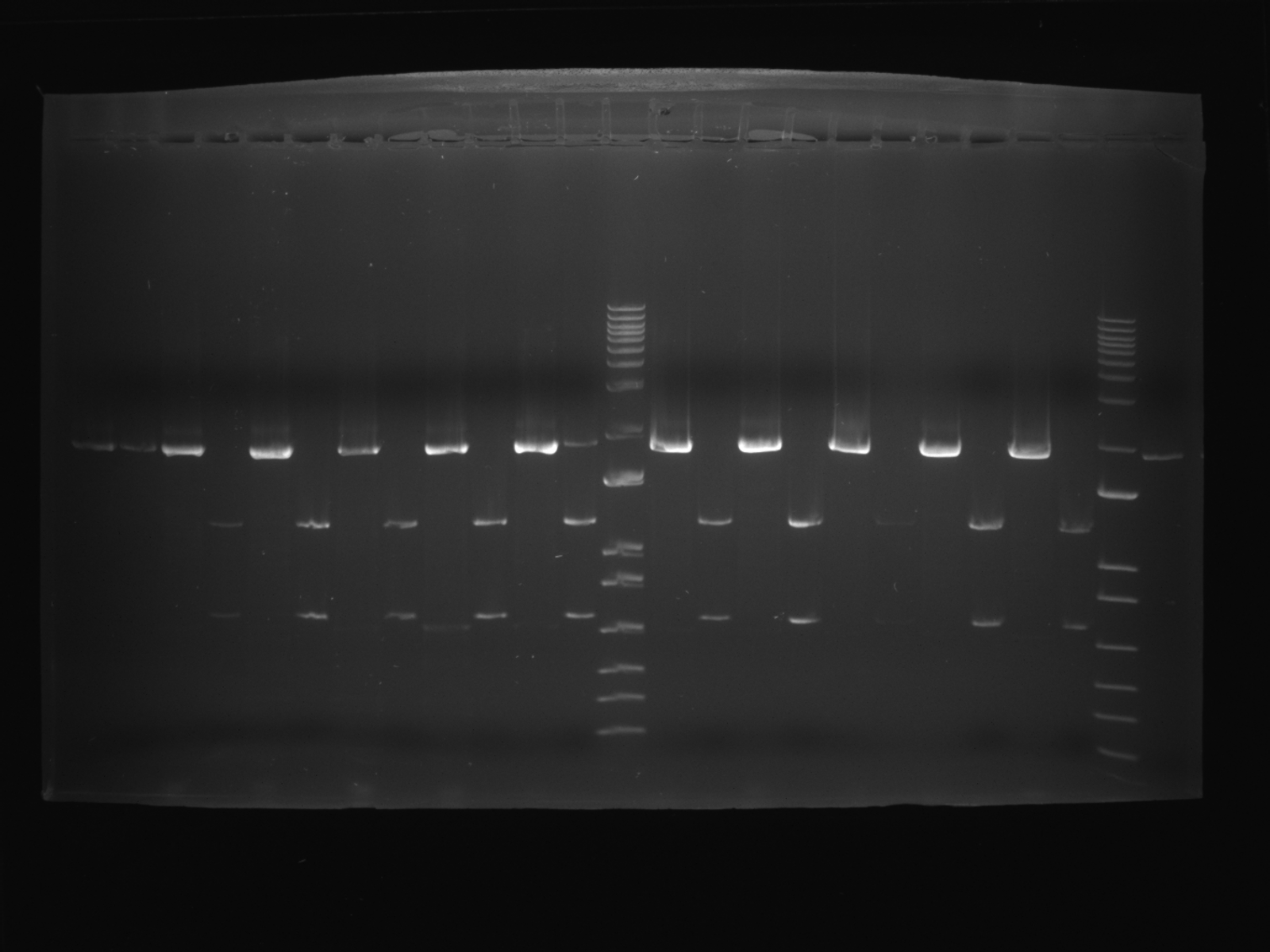

Supplement: Figure 3—figure supplement 1—source data 11. [file elife-75047-fig3-figsupp1-data11.zip › 75047Figure3S1SourceData11.Tif]

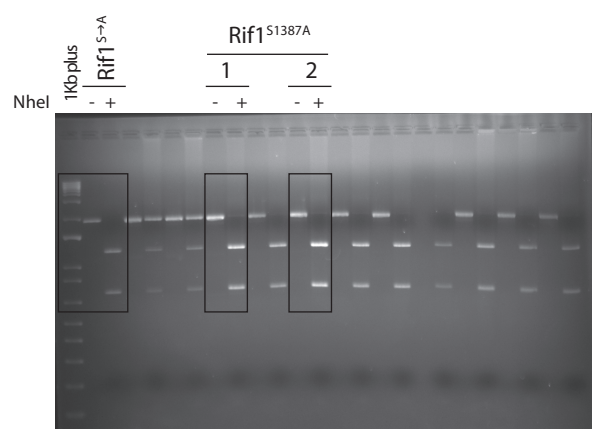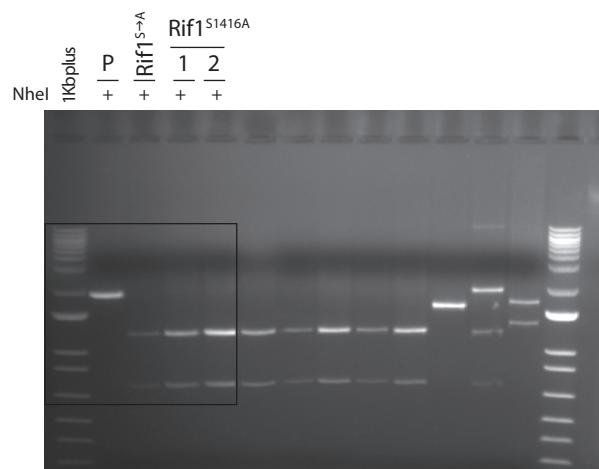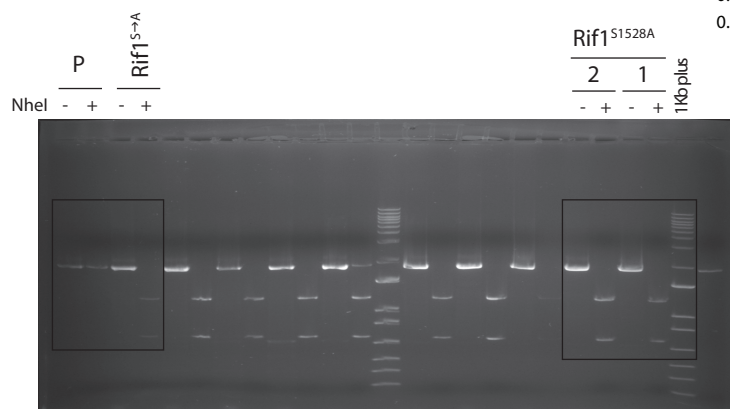

F

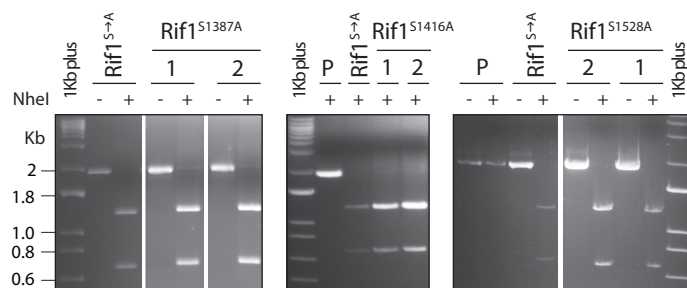

Figure 3 - figure supplement 1

Supplement: Figure 3—figure supplement 1—source data 12. [file elife-75047-fig3-figsupp1-data12.zip › 75047Figure3S1SourceData12.pdf]

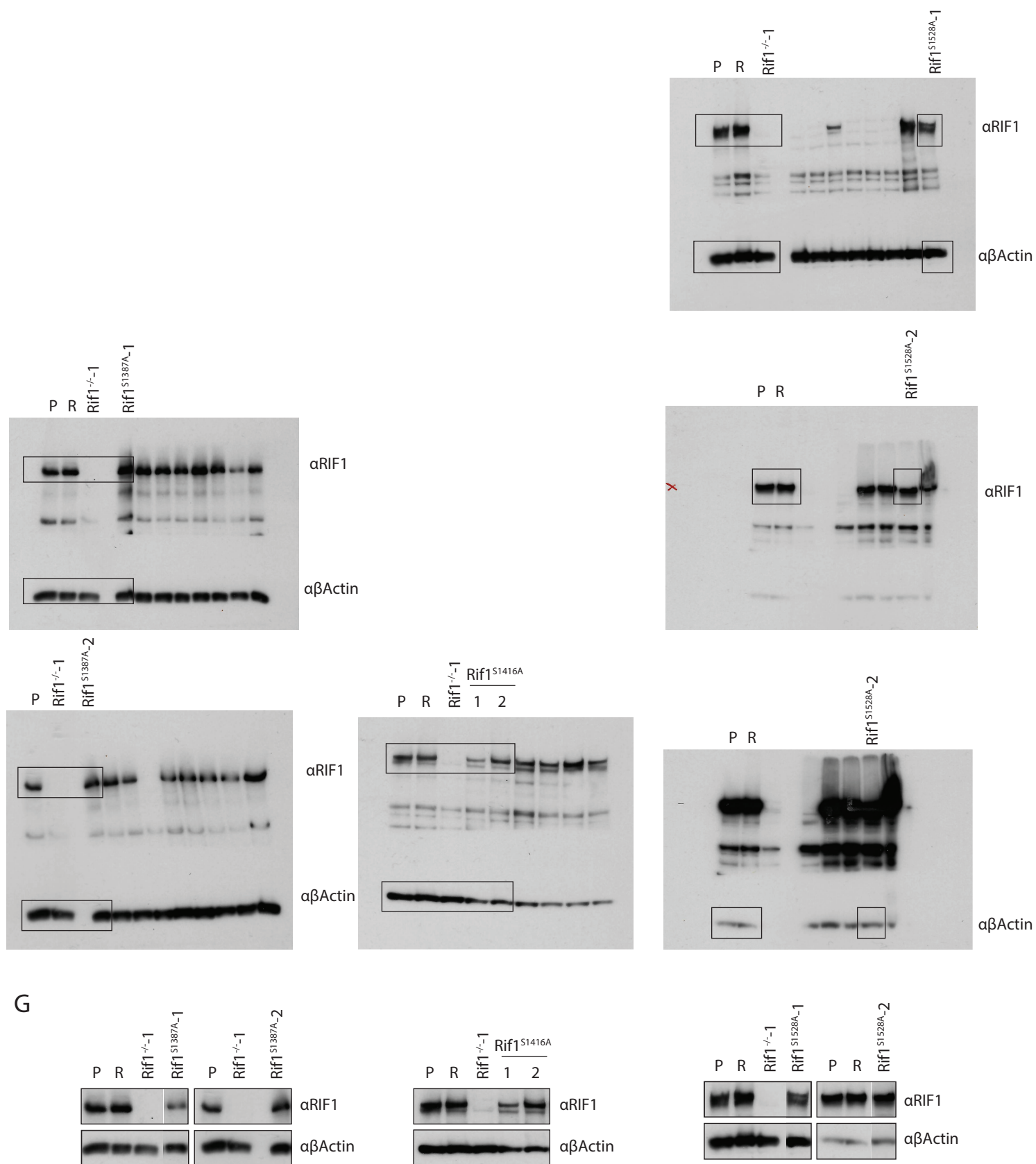

Figure 3 - figure supplement 1

Supplement: Figure 3—figure supplement 1—source data 18. [file elife-75047-fig3-figsupp1-data18.zip › 75047Figure3S1SourceData18.pdf]

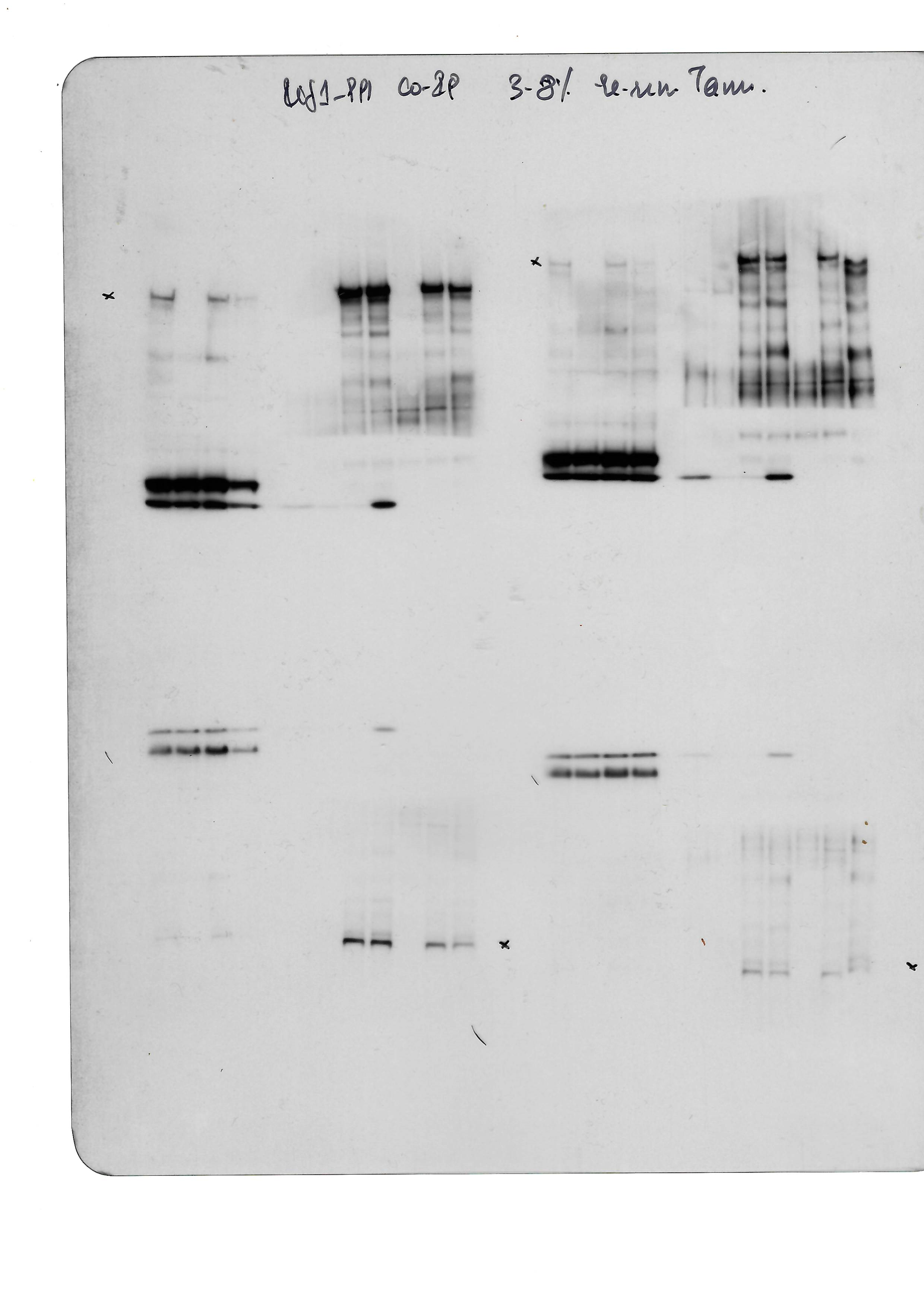

Supplement: Figure 4—source data 1. [file elife-75047-fig4-data1.zip › 75047Figure4SourceData1.tif]

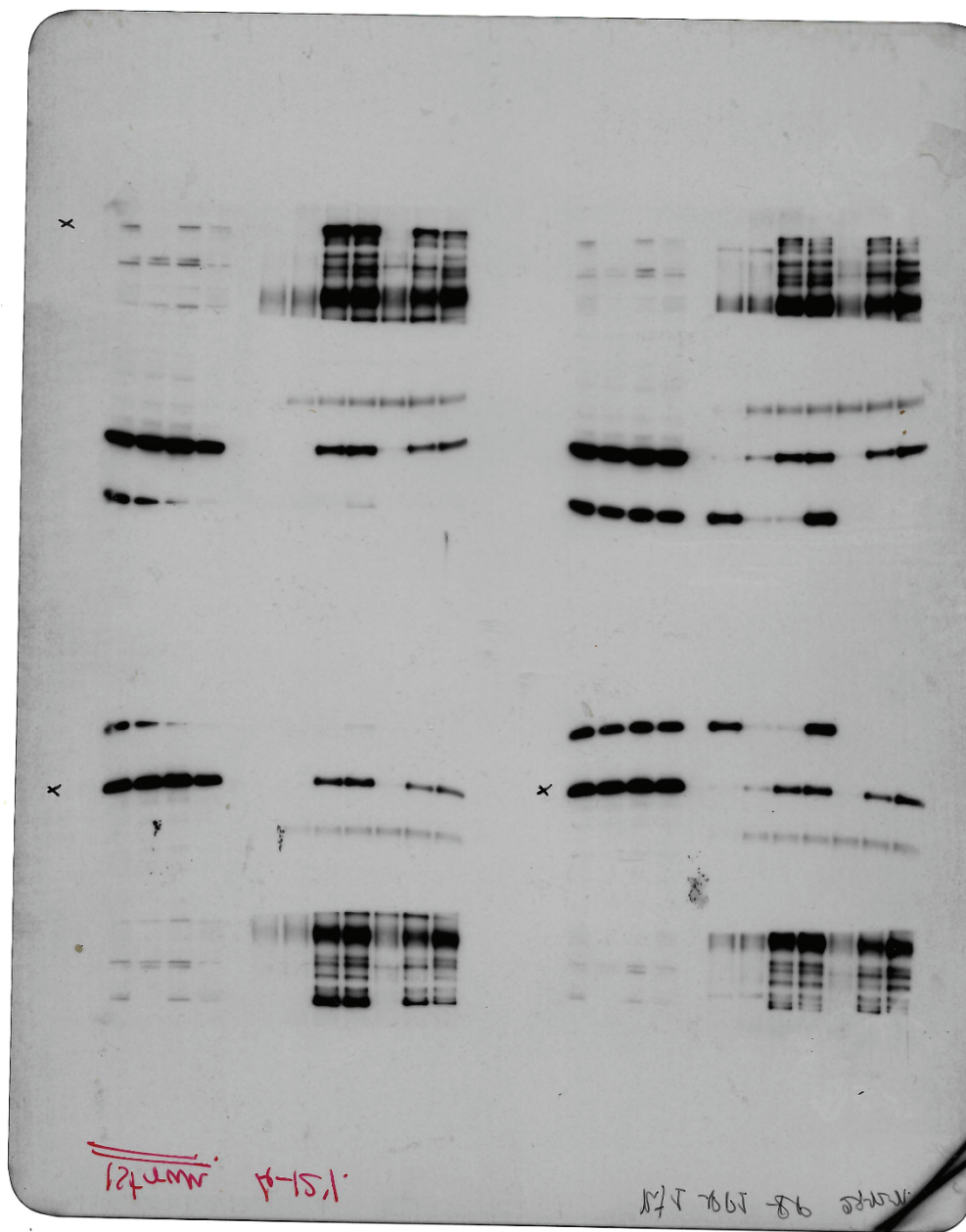

sample 98 1992 1/51

151-d. sample

Supplement: Figure 4—source data 3. [file elife-75047-fig4-data3.zip › 75047Figure4SourceData3.pdf]

**A**

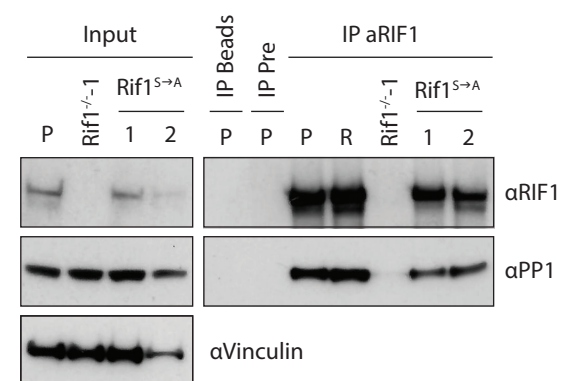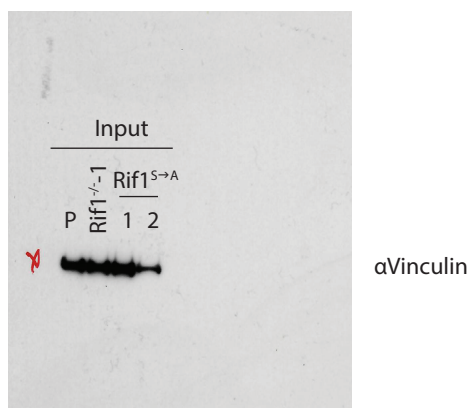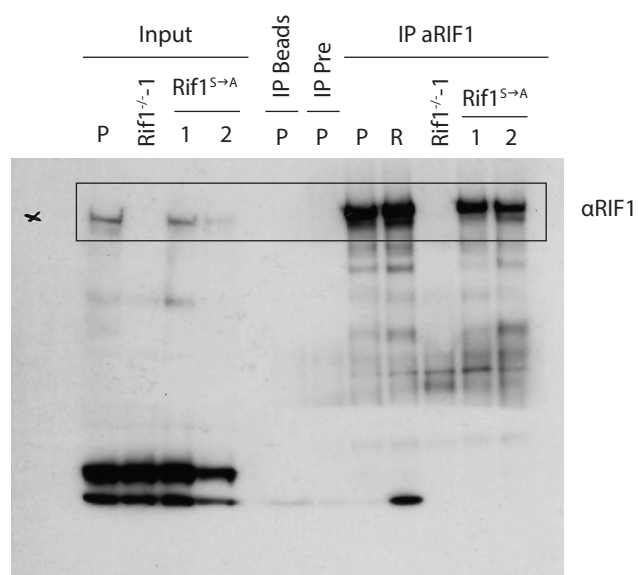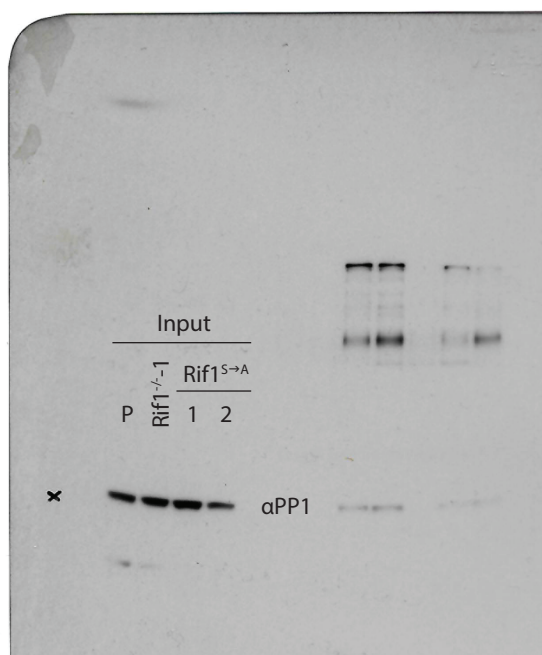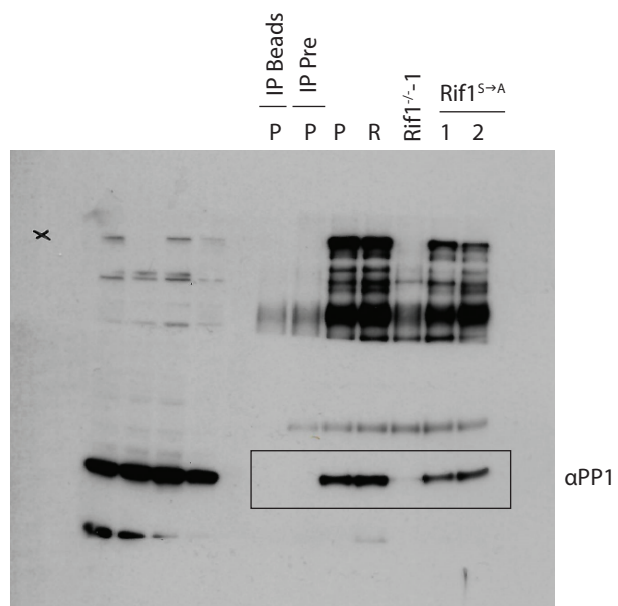

**Figure 4**

Supplement: Figure 4—source data 5. [file elife-75047-fig4-data5.zip › 75047Figure4SourceData5.pdf]

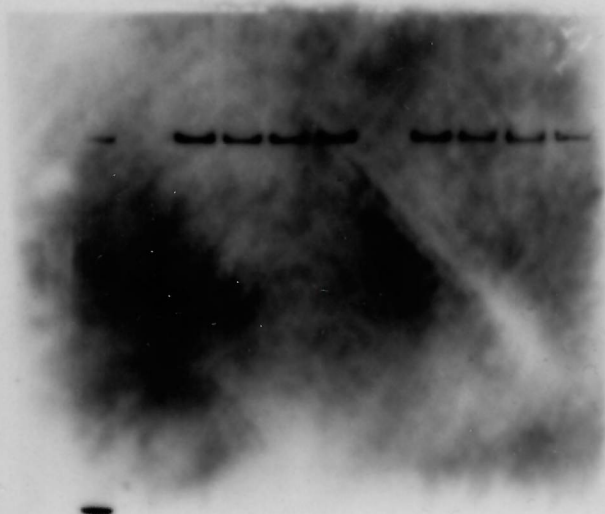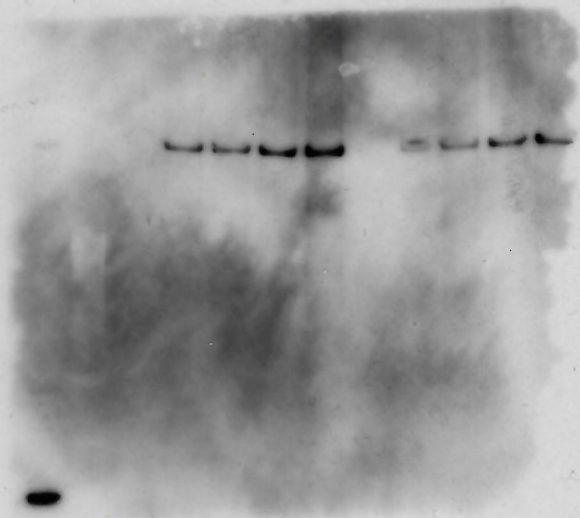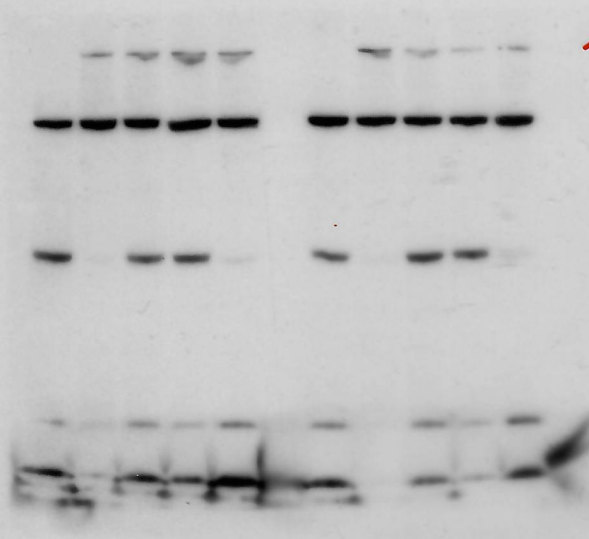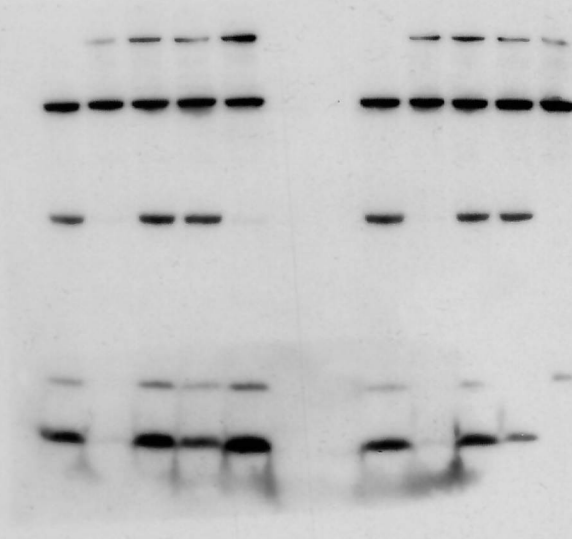

Supplement: Figure 4—source data 7. [file elife-75047-fig4-data7.zip › 75047Figure4SourceData7.pdf]
