# Supplementary material for: Protection of nascent DNA at stalled replication forks is mediated by phosphorylation of RIF1 intrinsically disordered region
Source: eLife. 2022 Apr 13;11:e75047. doi: 10.7554/eLife.75047 (PMC9007588; doi:10.7554/eLife.75047)
Supplement: Figure 2—source data 3. [file elife-75047-fig2-data3.zip › 75047Figure2SourceData3.pdf]

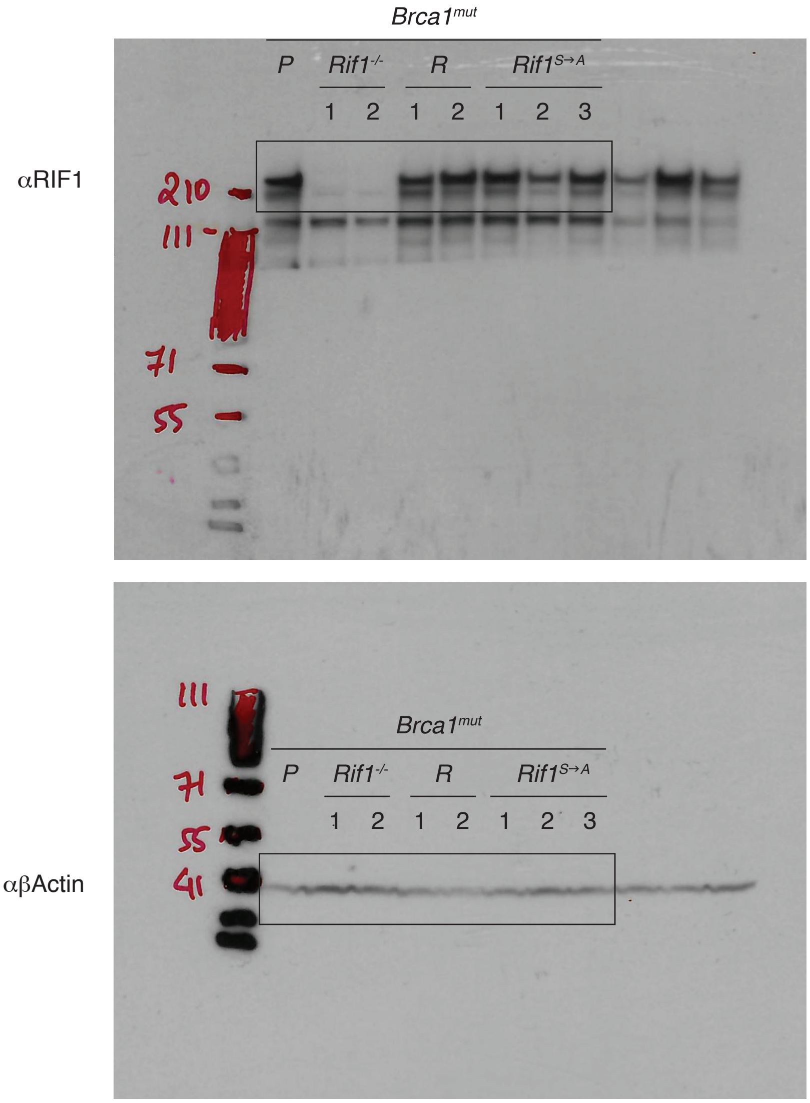

F

RIF1^WT VNEDSQAAAL----SCSDSQERES---TRRASQGLIS
RIF1^S→A VNEDAQAAAL----SCSDAQERES---TRRAAQGLIS

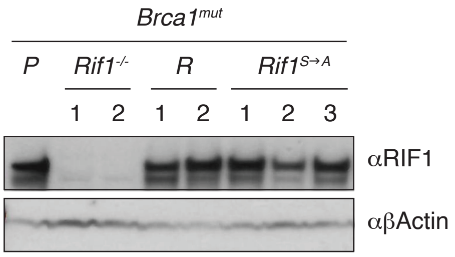

Figure 2
