# Supplementary material for: Protection of nascent DNA at stalled replication forks is mediated by phosphorylation of RIF1 intrinsically disordered region
Source: eLife. 2022 Apr 13;11:e75047. doi: 10.7554/eLife.75047 (PMC9007588; doi:10.7554/eLife.75047)
Supplement: Figure 4—source data 9. [file elife-75047-fig4-data9.zip › 75047Figure4SourceData9.pdf]

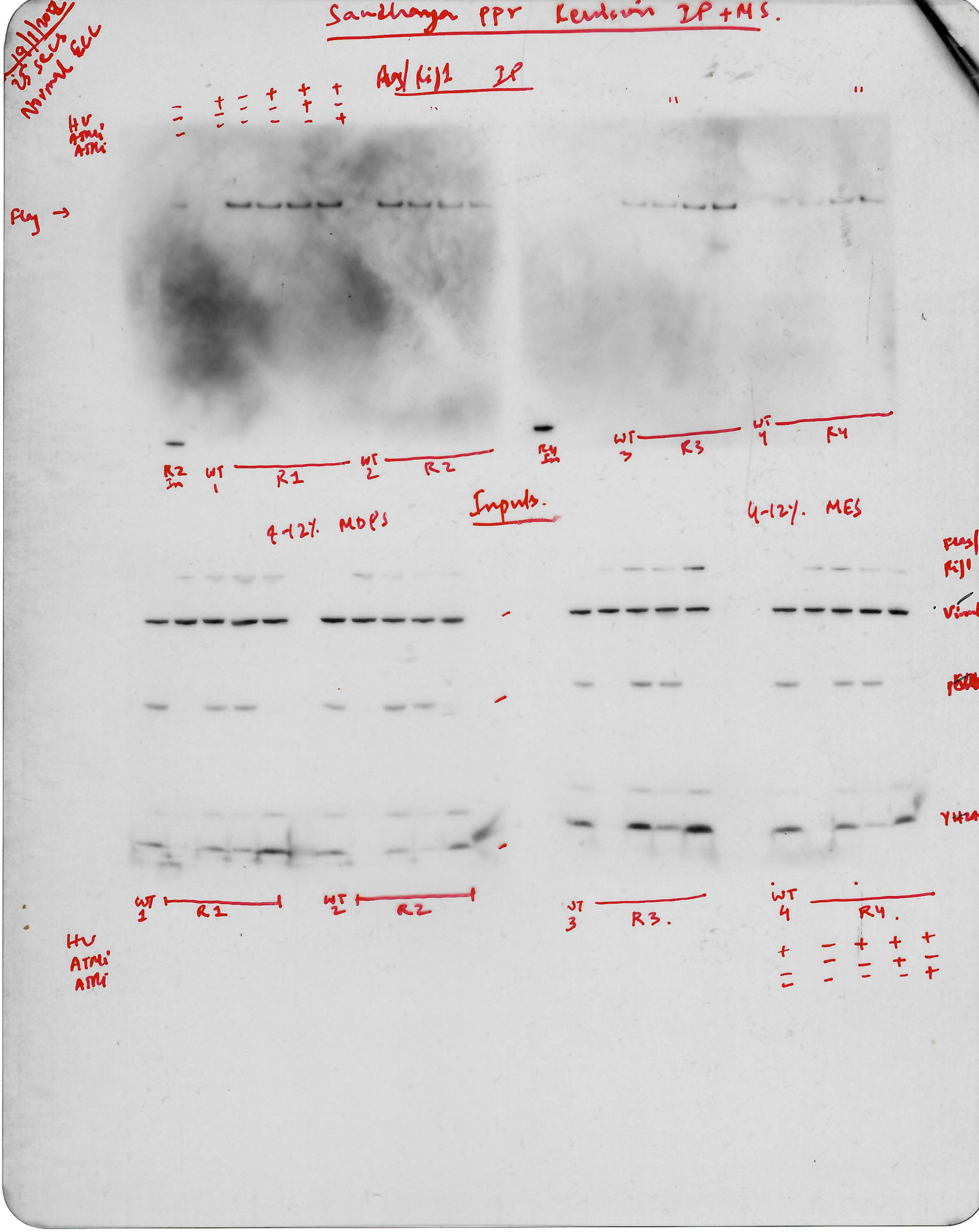
IP
25 secs
Normal ECL
HU
ATMi
ATRi
Flag →
R2 In
WT 1
R1
WT 2
R2
R4 In
WT 3
R3
WT 4
R4
Inputs.
4-12% MOPS
4-12% MES
WT 1
R1
WT 2
R2
WT 3
R3.
WT 4
R4.
HU
ATMi
ATRi
