# Supplementary material for: Protection of nascent DNA at stalled replication forks is mediated by phosphorylation of RIF1 intrinsically disordered region
Source: eLife. 2022 Apr 13;11:e75047. doi: 10.7554/eLife.75047 (PMC9007588; doi:10.7554/eLife.75047)
Supplement: Figure 4—source data 10. [file elife-75047-fig4-data10.zip › 75047Figure4SourceData10.pdf]

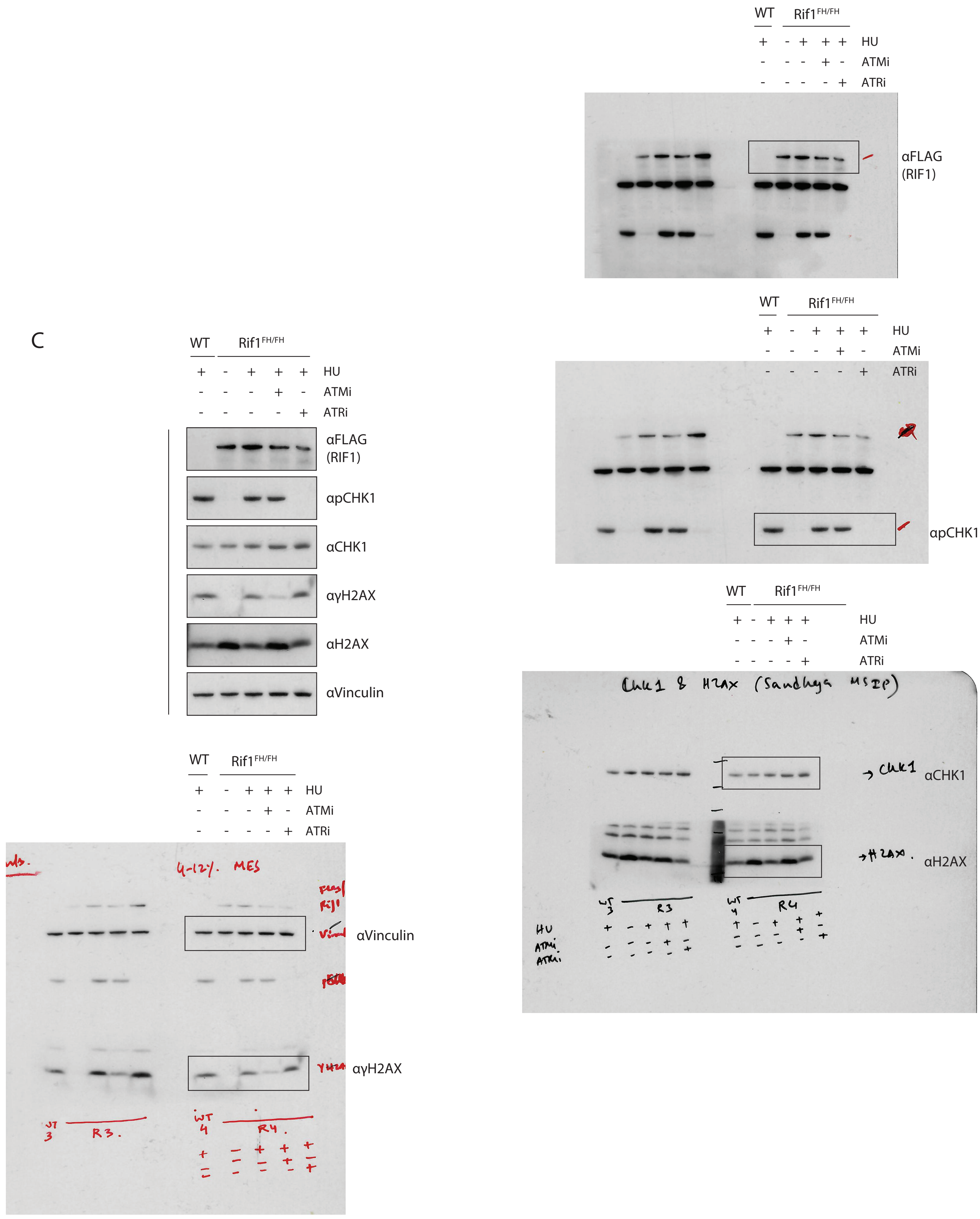

C
WT
Rif1 FH/FH
HU
ATMi
ATRi
αFLAG (RIF1)
αpCHK1
αCHK1
αγH2AX
αH2AX
αVinculin
Chk1 & H2AX (Sandhya MSIP)
4-12% MES

Figure 4
