# Supplementary material for: Protection of nascent DNA at stalled replication forks is mediated by phosphorylation of RIF1 intrinsically disordered region
Source: eLife. 2022 Apr 13;11:e75047. doi: 10.7554/eLife.75047 (PMC9007588; doi:10.7554/eLife.75047)
Supplement: Transparent reporting form [file elife-75047-transrepform1.docx]

***eLife’s* transparent reporting form**

We encourage authors to provide detailed information *within their submission* to facilitate the interpretation and replication of experiments. Authors can upload supporting documentation to indicate the use of appropriate reporting guidelines for health-related research (see [EQUATOR Network](http://www.equator-network.org/%20)), life science research (see the [BioSharing Information Resource](https://biosharing.org/)), or the [ARRIVE guidelines](http://www.plosbiology.org/article/info:doi/10.1371/journal.pbio.1000412) for reporting work involving animal research. Where applicable, authors should refer to any relevant reporting standards documents in this form.

If you have any questions, please consult our Journal Policies and/or contact us: [editorial@elifesciences.org](mailto:editorial@elifesciences.org).

**Sample-size estimation**

- You should state whether an appropriate sample size was computed when the study was being designed
- You should state the statistical method of sample size computation and any required assumptions
- If no explicit power analysis was used, you should describe how you decided what sample (replicate) size (number) to use

Please outline where this information can be found within the submission (e.g., sections or figure legends), or explain why this information doesn’t apply to your submission:

The sample-size estimation for number of replicates per experiment and number of scored events within each experiment (for metaphase and fiber assays) was calculated based on similar experiments performed in published work and from experience with the employed assays/techniques/protocols.

**Replicates**

- You should report how often each experiment was performed
- You should include a definition of biological versus technical replication
- The data obtained should be provided and sufficient information should be provided to indicate the number of independent biological and/or technical replicates
- If you encountered any outliers, you should describe how these were handled
- Criteria for exclusion/inclusion of data should be clearly stated
- High-throughput sequence data should be uploaded before submission, with a private link for reviewers provided (these are available from both GEO and ArrayExpress)

Please outline where this information can be found within the submission (e.g., sections or figure legends), or explain why this information doesn’t apply to your submission:

This information can be found in the Figure legends.

**Statistical reporting**

- Statistical analysis methods should be described and justified
- Raw data should be presented in figures whenever informative to do so (typically when N per group is less than 10)
- For each experiment, you should identify the statistical tests used, exact values of N, definitions of center, methods of multiple test correction, and dispersion and precision measures (e.g., mean, median, SD, SEM, confidence intervals; and, for the major substantive results, a measure of effect size (e.g., Pearson's r, Cohen's d)
- Report exact p-values wherever possible alongside the summary statistics and 95% confidence intervals. These should be reported for all key questions and not only when the p-value is less than 0.05.

Please outline where this information can be found within the submission (e.g., sections or figure legends), or explain why this information doesn’t apply to your submission:

This information can be found in the Figure legends and in the Statistical analysis subsection of Materials & Methods.

(For large datasets, or papers with a very large number of statistical tests, you may upload a single table file with tests, Ns, etc., with reference to sections in the manuscript.)

**Group allocation**

- Indicate how samples were allocated into experimental groups (in the case of clinical studies, please specify allocation to treatment method); if randomization was used, please also state if restricted randomization was applied
- Indicate if masking was used during group allocation, data collection and/or data analysis

Please outline where this information can be found within the submission (e.g., sections or figure legends), or explain why this information doesn’t apply to your submission:

Group allocation is not relevant to this study.

**Additional data files (“source data”)**

- We encourage you to upload relevant additional data files, such as numerical data that are represented as a graph in a figure, or as a summary table
- Where provided, these should be in the most useful format, and they can be uploaded as “Source data” files linked to a main figure or table
- Include model definition files including the full list of parameters used
- Include code used for data analysis (e.g., R, MatLab)
- Avoid stating that data files are “available upon request”

Please indicate the figures or tables for which source data files have been provided:

Source Data files have been provided for: Figure 1 panel A (Figure 1-source data 1.docx and Figure 1-source data 2.docx); Figure 1 panel C (Figure 1-source data 3.tiff and Figure 1-source data 3 with labels.pdf); Figure 1 panel D (Figure 1-source data 4.xlsx); Figure 2 panel F (Figure 2-source data 1.tiff, Figure 2-source data 2.tiff, and Figure 2-source data 1 and 2 with labels.pdf); Figure 3 panel A (Figure 3-source data 1-right side.tiff and Figure 3-source data 1 with labels.pdf); Figure 3 panel C (Figure 3-source data 2.tif, Figure 3-source data 3.tif, Figure 3-source data 4.tif, Figure 3-source data 5.tif); Figure 4 panel A (Figure 4- source data 1-left side.tif, Figure 4- source data 2-left side.tif, Figure 4- source data 3-left side.pdf, Figure 4- source data 4-left side.tif, Figure 4-source data 1,2,3 and 4 with labels.pdf), Figure 1-figure supplement 1 panel C (Figure 1-figure supplement 1 -source data 1.tif, Figure 1-figure supplement 1 -source data 2.tif, Figure 1-figure supplement 1 -source data 3.tif, Figure 1-figure supplement 1 -source data 4.tif); Figure 2-figure supplement 1 panel C( Figure 2-figure supplement 1 2-source data 1.jpg, Figure 2-figure supplement 1 2-source data 1 with labels.pdf); Figure 2-figure supplement 1 panel H (Figure 2- figure supplement 1- source data 2.tif, Figure 2- figure supplement 1- source data 2 with labels.pdf); Figure 2-figure supplement 1 panel I (Figure 2- figure supplement 1- source data 3-right side.tif, Figure 2- figure supplement 1- source data 4-right side.tif, Figure 2- figure supplement 1- source data 5-right side.tif, Figure 2-figure supplement 1- source data 3,4 and 5 with labels.pdf); Figure 3-figure supplement 1 panel B (Figure 3-figure supplement 1 3-source data 1-left side.tif, Figure 3-figure supplement 1 3-source data 1 with labels.pdf); Figure 3-figure supplement 1 panel C (Figure 3- figure supplement 1- source data 2.tif, Figure 3- figure supplement 1- source data 3.tif, Figure 3- figure supplement 1- source data 2 and 3 with labels.pdf); Figure 3, figure supplement 1 panel E ( Figure 3- figure supplement 1- source data 4-left and right side below.tif, Figure 3- figure supplement 1- source data 5-left side below.tif, Figure 3-figure supplement 1 -source data 4 and 5 with labels.pdf); Figure 3 figure supplement 1 panel F (Figure 3- figure supplement 1- source data 6.tif, Figure 3- figure supplement 1- source data 7.tif, Figure 3- figure supplement 1- source data 8.tif, Figure 3-figure supplement 1 -source data 6, 7 and 8 with labels.pdf); Figure 3-figure supplement 1 panel G (Figure 3- figure supplement 1- source data 9.tif, Figure 3- figure supplement 1- source data 10-top.tif, Figure 3- figure supplement 1- source data 11-below.tif, Figure 3- figure supplement 1- source data 12-below.tif, Figure 3- figure supplement 1- source data 13-below.tif, Figure 3-figure supplement 1 -source data 9,10,11,12 and 13 with labels.pdf). The list of Source Data files (with legends) has been included at the end of the manuscript text file.
